# Directed evolution-based discovery of ligands for in vivo restimulation of chimeric antigen receptor T cells

**DOI:** 10.1038/s41551-025-01470-0

**Published:** 2025-08-25

**Authors:** Tomasz M. Grzywa, Alexandra Neeser, Ranjani Ramasubramanian, Anna Romanov, Ryan Tannir, Naveen K. Mehta, Benjamin Cossette, Duncan M. Morgan, Beatriz Goncalves, Ina Sukaj, Elisa Bergaggio, Stephan Kadauke, Regina M. Myers, Luca Paruzzo, Guido Ghilardi, Austin Cozzone, Stephen J. Schuster, Noelle Frey, Libin Zhang, Parisa Yousefpour, Wuhbet Abraham, Heikyung Suh, Marco Ruella, Stephan A. Grupp, Roberto Chiarle, K. Dane Wittrup, Leyuan Ma, Darrell J. Irvine

**Affiliations:** 1 The Raymond G. Perelman Center for Cellular and Molecular Therapeutics, Children’s Hospital of Philadelphia, Philadelphia, PA USA; 2 Department of Bioengineering, School of Engineering and Applied Science, University of Pennsylvania, Philadelphia, PA USA; 3 David H. Koch Institute for Integrative Cancer Research, MIT, Cambridge, MA USA; 4 Department of Biological Engineering, MIT, Cambridge, MA USA; 5 College of Arts and Sciences, University of Pennsylvania, Philadelphia, PA USA; 6 Department of Chemical Engineering, MIT, Cambridge, MA USA; 7 Department of Cancer Biology, Perelman School of Medicine, University of Pennsylvania, Philadelphia, PA USA; 8 Department of Pathology, Boston Children’s Hospital and Harvard Medical School, Boston, MA USA; 9 Department of Pathology and Laboratory Medicine, Perelman School of Medicine, University of Pennsylvania, Philadelphia, PA USA; 10 Division of Oncology, Department of Pediatrics, Perelman School of Medicine, University of Pennsylvania, Philadelphia, PA USA; 11 Cellular Therapy and Transplant Section and Cancer Immunotherapy Program, Children’s Hospital of Philadelphia, Philadelphia, PA USA; 12 Center for Cellular Immunotherapies, University of Pennsylvania, Philadelphia, PA USA; 13 Division of Hematology/Oncology, University of Pennsylvania Perelman School of Medicine, Philadelphia, PA USA; 14 Abramson Cancer Center, University of Pennsylvania, Philadelphia, PA USA; 15 Department of Molecular Biotechnology and Health Sciences, University of Torino, Torino, Italy; 16 Department of Materials Science and Engineering, MIT, Cambridge, MA USA; 17 Ragon Institute of Massachusetts General Hospital, Cambridge, MA USA; 18 Howard Hughes Medical Institute, Chevy Chase, MD USA; 19 Present Address: Department of Immunology and Microbiology, The Scripps Research Institute, La Jolla, CA USA

**Keywords:** Biotechnology, Vaccines, Immunotherapy

## Abstract

Chimeric antigen receptor (CAR) T cell therapy targeting CD19 elicits remarkable clinical efficacy in B cell malignancies, but many patients relapse owing to failed expansion and/or progressive loss of CAR-T cells. We recently reported a strategy to potently restimulate CAR-T cells in vivo, enhancing their functionality by administration of a vaccine-like stimulus comprised of surrogate peptide ligands for a CAR linked to a lymph node-targeting amphiphilic PEG-lipid (amph-vax). Here we demonstrate a general strategy to discover and optimize peptide mimotopes enabling amph-vax generation for any CAR. We use yeast surface display to identify peptide binders to FMC63 (the scFv used in clinical CD19 CARs), which are then subsequently affinity matured by directed evolution. CAR-T vaccines using these optimized mimotopes triggered marked expansion and memory development of CD19 CAR-T cells in both syngeneic and humanized mouse models of B-acute lymphoblastic leukaemia/lymphoma, and enhanced control of disease progression compared with CD19 CAR-T-only-treated mice. This approach enables amph-vax boosting to be applied to any clinically relevant CAR-T cell product.

## Abstract (web summary)

An amphiphilic mimotope vaccine can be used in tandem with Food and Drug Administration (FDA)-approved CD19 CAR-T cell products.

## Main

CD19-targeted chimeric antigen receptor (CAR) T cell therapy elicits anti-tumour activity in the treatment of relapsed or refractory B cell acute lymphoblastic leukaemia (B-ALL) and lymphoma in both paediatric and adult patients^1,2^. This clinical success has led to the US Food and Drug Administration (FDA) approval of four CD19-targeted CAR-T products, namely TECARTUS, KYMRIAH, YESCARTA and BREYANZI^1^. However, a substantial fraction (30–60%) of patients still experience leukaemia relapse, among which ~50% are CD19-positive relapses^3^, suggesting impaired function and/or persistence of the infused CAR-T cells. Robust CAR-T cell engraftment and expansion in vivo is a prerequisite for anti-tumour efficacy^4^, a feature that is affected by several characteristics of the CAR-T cell product such as T cell quality^5^, CAR design^3,4,6^ and the proportion of early memory T cells in the product^7^. In current CAR-T cell therapy treatment regimens, patients usually receive lymphodepletion to promote CAR-T cell engraftment^8^. Interestingly, multiple reports have shown a correlation between the early-stage expansion of CAR-T cells and initial tumour burden^8,9^. These findings suggest that the capacity of CAR-T cells to receive antigen-specific stimulation post-infusion is critical for CAR-T cell expansion, engraftment and potentially long-term persistence. However, whether the antigens presented by tumour cells are sufficient to optimally stimulate CAR-T cells remains unclear.

Vaccination is a natural process whereby T cells receive antigen-specific stimulation leading to proliferation, differentiation and induction of effector functions^10^—effects that could be beneficial in promoting CAR-T cell function in vivo. However, traditional vaccines provide antigens that must be processed into peptides by professional antigen-presenting cells (APCs) and presented on major histocompatibility complex (MHC) molecules to stimulate T cells through the T cell receptor (TCR). This is challenging to adapt to the setting of engineered CAR-T cells, as current CAR-T products are polyclonal and express uncharacterized TCRs. Further, recent next-generation engineering efforts such as knocking CARs into the TCR locus produce CAR-T lacking endogenous TCRs^11^. Proof-of-concept demonstration of CAR-T cells generated from virus-specific T cells followed by vaccine boosting using the viral-specific TCR has been shown in small clinical trials, yet the benefits observed with this approach have been limited^12,13^.

**Extended Data Fig. 1:**
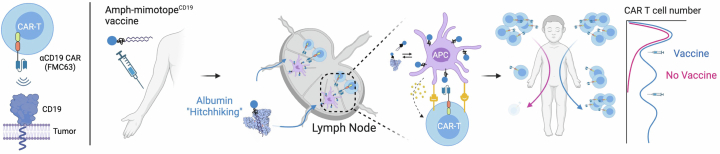
The concept of designing an amph-mimotope vaccine for CD19 CAR-T cell therapy. Created in BioRender. Ma, L. (2025) https://BioRender.com/miyfij7.

To overcome these issues, we recently developed a molecularly targeted amphiphilic vaccine (amph-vax) approach to directly stimulate CAR-T cells through the CAR^14,15^. Amph-vax molecules were generated by linking a CAR-ligand to albumin-binding phospholipid polymers. Following parenteral injection, these CAR-ligand conjugates associate with endogenous albumin present in interstitial fluid and are carried into lymph and downstream draining lymph nodes (dLNs). In the dLNs, the lipid tails of amph-vax ligands insert into cell membranes, including membranes of professional APCs, allowing APCs to directly ‘present’ the CAR ligand to CAR-T cells together with additional co-stimulation and cytokine support, making them de novo CAR-T cell-priming APCs^14^ (Extended Data Fig. 1). Amph-vax stimulation led to pronounced CAR-T cell expansion with enhanced functionality, memory formation and substantially enhanced tumour clearance^14,15^. We also found that vaccine boosting of CAR-T cells promotes antigen spreading and the induction of endogenous anti-tumour immune responses, which prevent antigen loss-mediated tumour escape^15^. We demonstrated these effects using CARs that recognize synthetic ligands (for example, fluorescein) and a model EGFRvIII-specific CAR, where the scFv domain of the chimeric receptor recognized a linear peptide epitope in EGFRvIII that is readily synthesized as an amph-ligand. However, to generalize this strategy and enable amph-vax boosting with any arbitrary CAR of interest, an approach is needed for de novo generation of surrogate peptide ligands for CARs.

Here we demonstrate a workflow to identify peptide mimotope ligands for CAR receptors using yeast surface display-based directed evolution^16^. We developed the pipeline by discovering an amph-ligand that can be used with all four FDA-approved CD19 CAR-T cell therapies. We created a customized mimotope yeast library to identify peptides that bind to the licensed CD19 CARs. Affinity maturation of hits from this library led to the identification of a peptide with high affinity for the CAR. An amphiphile-mimotope (amph-mimotope) vaccine based on this peptide efficiently stimulated CD19 CAR-T cells in vitro, triggered their expansion in vivo and enhanced clearance of leukaemia in both syngeneic and humanized mouse models of B-ALL/lymphoma. To show generality, we also generated amph-mimotopes for a second human CAR recognizing the anaplastic lymphoma kinase (ALK) antigen and a murine CD19 CAR. Collectively, these results show the successful development of a clinically translatable amph-mimotope vaccine with the potential for endowing current FDA-approved CD19 CAR-T cell therapies with enhanced anti-leukaemic activity.

## Results

### Antigen^+^ tumour cells drive transient CD19 CAR-T expansion

**Fig. 1:**
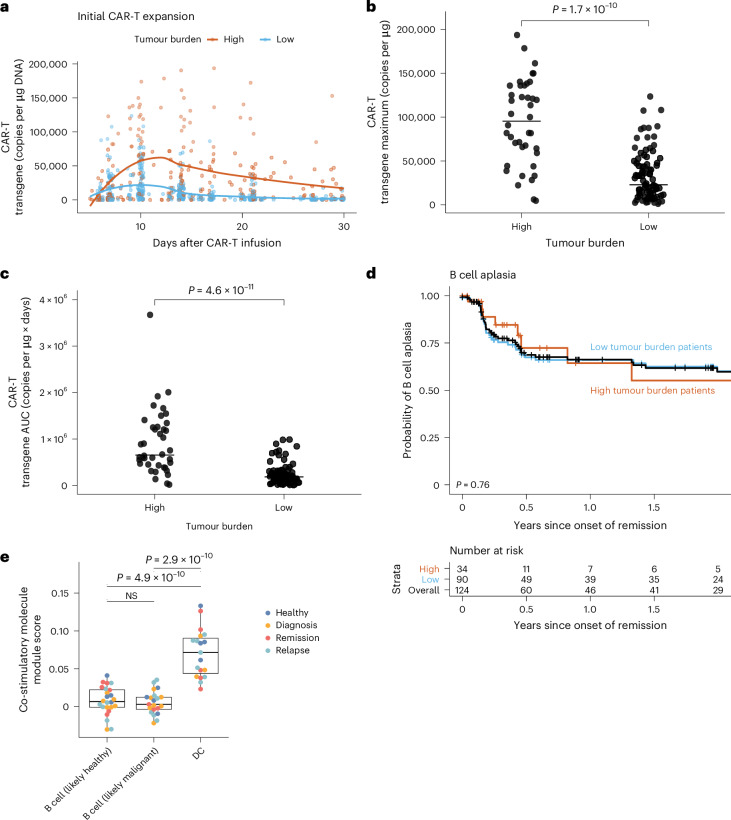
CD19 CAR-T cell expansion and B cell aplasia in patients with paediatric B-ALL with initial high or low tumour burden, and rationale for a DC-targeting CD19 CAR stimulating vaccine. **a**, Scatterplot of peripherally circulating CAR-T cells in patients with paediatric B-ALL with initially high (≥40%, *n* = 40) or low tumour burden (<40%, *n* = 90) measured by qPCR over time during the first month after infusion. Solid lines show trends generated by locally estimated scatterplot smoothing. The median time to peak CAR-T expansion was 10 days after infusion in both cohorts. **b**,**c**, Maximum concentration (**b**) and area under the curve (AUC) (**c**) of CAR-transgene levels measured by qPCR, broken down by tumour burden. ***P < 0.001, by two-sided Wilcoxon rank-sum tests (*n* = 40 for high tumour burden, *n* = 90 for low tumour burden for **b** and **c**). **d**, Kaplan–Meier analysis of B cell aplasia for all patients (black) or patients with high (orange) or low (blue) tumour burden. B cell aplasia is defined as the time to the emergence of ≥1% CD19-positive B cell in bone marrow aspirate or ≥3% B cell by peripheral blood flow cytometry or CD19-positive relapse. Data were censored for patients who had CD19-negative relapse, reinfusion for hematogones in the marrow, alternative therapy including other CAR-T therapy or hematopoietic stem cell transplant, or non-relapse mortality. The probability of continued B cell aplasia at 2 years was similar in both cohorts (*P* = 0.76 by log-rank test). At 6 months, it was 72% (95% CI 55–95) in high and 67% (95% CI 57–78) in low tumour burden patients. **e**, Box plot of co-stimulatory molecule module scores expressed by B cells (likely healthy (*n* = 24) and likely malignant (*n* = 21)) and DC (*n* = 19). The box plots show median, 25th percentile and 75th percentile. The whiskers extend from each hinge to the most extreme value within 1.5 interquartile range (IQR). Data beyond this range are plotted individually. Each point represents the average of all cells of that phenotype within a patient, by a two-sided Wilcoxon rank-sum test. NS, not significant. Source data

**Extended Data Fig. 2:**
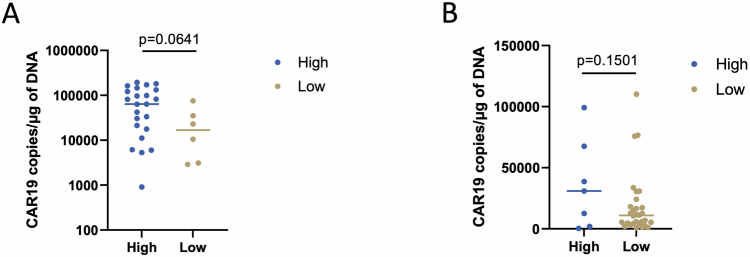
CD19 CAR-T cell expansion in Adult B-ALL and B-cell lymphoma patients with initial high or low tumor burden. (**a**) The impact of high tumor burden on CAR-T expansion in a cohort of relapsed/refractory ALL patients (n = 29) treated with 4-1BB CAR-T19 (CTL019; NCT02030847). High tumor burden was defined as bone marrow involvement before CART19 higher than 5%. The majority of patients (23/29, 79.3%) had a high disease burden before treatment. High tumor burden correlated with higher peak of expansion (CAR19 copies/μg of DNA: high tumor burden: 77,109 vs. low tumor burden: 25,064; p = 0.0641;). P-values are calculated with a two-sided unpaired student’s t-test. (**b**) The impact of high tumor burden on CAR-T expansion in a cohort of relapsed/refractory B-NHL patients treated with 4-1BB CAR-T19 CTL019 within a clinical trial (n = 37; NCT02030834). High tumor burden was defined as serum LDH higher than 1.5 folds the upper normal limit at infusion. In this cohort, we observed a trend toward higher CAR-T19 expansion in patients with high disease burden (n = 7, 18.9%) than in patients with low tumor burden (n = 30, 81.1%) (CAR19 copies/μg of DNA 35,857 vs 18,718 respectively; p = 0.1501). P-values are calculated with a two-sided unpaired student’s t-test. Source data

CD19-positive relapse is often associated with limited CAR-T persistence and/or impaired functionality^3,17^. To provide insight into whether the level of available antigen stimulation impacts early CAR-T expansion/persistence, we analysed data from several early clinical trials in children and young adults with B-ALL from the Children’s Hospital of Philadelphia (NCT01626495 (ref. ^18^) and NCT02906371 (ref. ^9^)) and in adult patients with B-ALL (NCT02030847 (ref. ^19^)) or B cell lymphoma (NCT02030834 (ref. ^20^)) from the University of Pennsylvania. We found that CAR-T cell expansion is generally associated with initial tumour burden, with CAR-T cells infused into patients with high tumour burden (>40% bone marrow blasts in pre-infusion bone marrow aspirate) exhibiting more pronounced expansion and longer persistence (Fig. 1a–c and Extended Data Fig. 2a,b). This association is statistically significant in children and young adults with B-ALL (Fig. 1a–c) and exhibits a trend towards significance in adult B-ALL and B cell lymphoma (Extended Data Fig. 2a,b), consistent with observations from earlier studies^8,9^.

**Extended Data Fig. 3:**
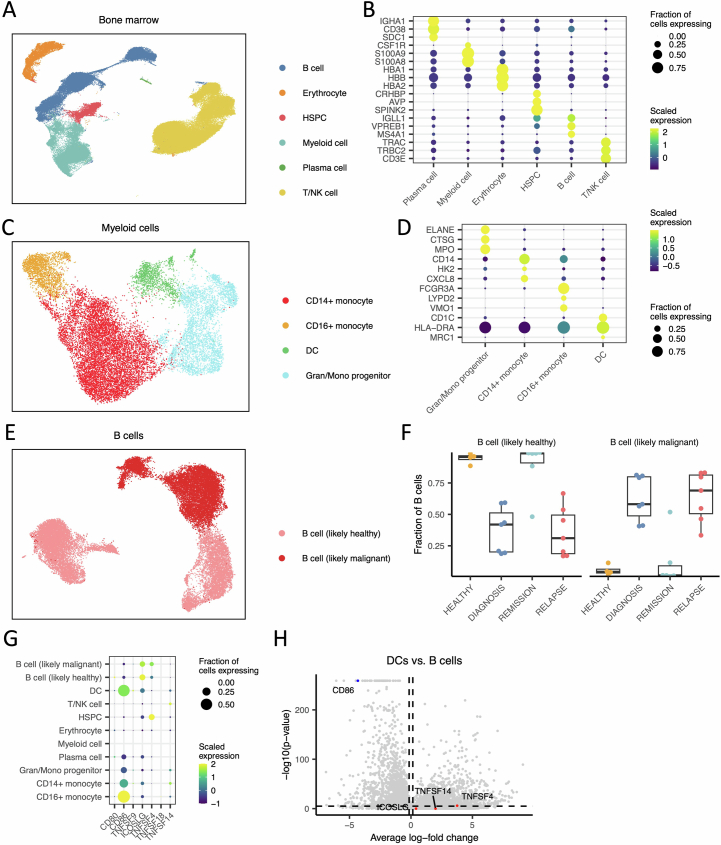
Single-cell RNA-seq analysis of immune cells from B-ALL patients. (**a**) UMAP of single cells isolated from the bone marrow of human B-ALL patients, colored by cell phenotype. (**b**) Dot plot showing the scaled expression and fraction of cells expressing marker genes for each cell phenotype. (**c**) UMAP of myeloid cells, colored by cell phenotype. (**d**) Dot plot showing the scaled expression and fraction of cells expressing marker genes associated with each myeloid cell phenotype. (**e**) UMAP of B cells, with likely-healthy and likely-malignant cells distinguished. (**f**) Frequency of likely-healthy and likely-malignant B cells in samples with each diagnosis (n = 4 for healthy; n = 7 for diagnosis; n = 6 for remission; n = 7 for relapse). The boxplots show median, 25th percentile, and 75th percentiles. The whiskers extend from each hinge to the most extreme value within 1.5 IQR. Data beyond this range is plotted individually. (**g**) Dot plot showing the scaled expression and fraction of cells expressing each co-stimulatory receptor. (**h**) Volcano plot of genes differentially expressed between DCs and B cells. P-values are calculated with a two-sided Wilcoxon rank-sum test and are adjusted using Bonferroni correction. Source data

Since CD19 CAR-T cells target normal B cells, continued B cell aplasia can be used as a pharmacodynamic measure for the presence of functional CD19 CAR-T cells^18^. Nearly half of the patients with paediatric B-ALL receiving CD19 CAR-T cell therapy experienced B cell recovery after initial B cell aplasia (Fig. 1d), indicating loss of functional CAR-T cells. Interestingly, there was no significant association between the initial tumour burden and the probability of B cell recovery, suggesting that increased early stimulation from tumour cells was not sufficient to maintain CAR-T function. A recent long-term follow-up study found that a higher ratio of peak CAR-T expansion to tumour burden correlates with overall survival and is a better prediction of long-term survival than the absolute magnitude of T cell expansion or tumour burden^21^. These data suggest that if CAR-T cells receive greater antigen stimulation following infusion, they can undergo greater expansion, an important correlate of response to therapy^21,22^. However, sustained anti-tumour function over time requires that T cells receive restimulation by professional APCs, particularly dendritic cells (DCs), which express high levels of co-stimulatory receptors and cytokines that are lacking from tumour cells and resting B cells^23,24^. By mining a published dataset^25^, we observed significantly higher co-stimulatory molecule expression in DCs than healthy and likely malignant B cells in patients with B-ALL (Fig. 1e and Extended Data Fig. 3a–h). We previously also observed that the comprehensive suite of co-stimulatory molecules expressed by DCs is essential for promoting memory differentiation and optimal amplification of CAR-T cells during vaccine boosting^14^. These observations suggested to us that an efficient and safe DC-targeted vaccination approach to reinvigorate and sustain CAR-T cell function could be impactful for enhancing current clinical CD19-targeting CAR-T cell therapies.

### Discovery of CAR peptide ligands using yeast surface display

**Fig. 2:**
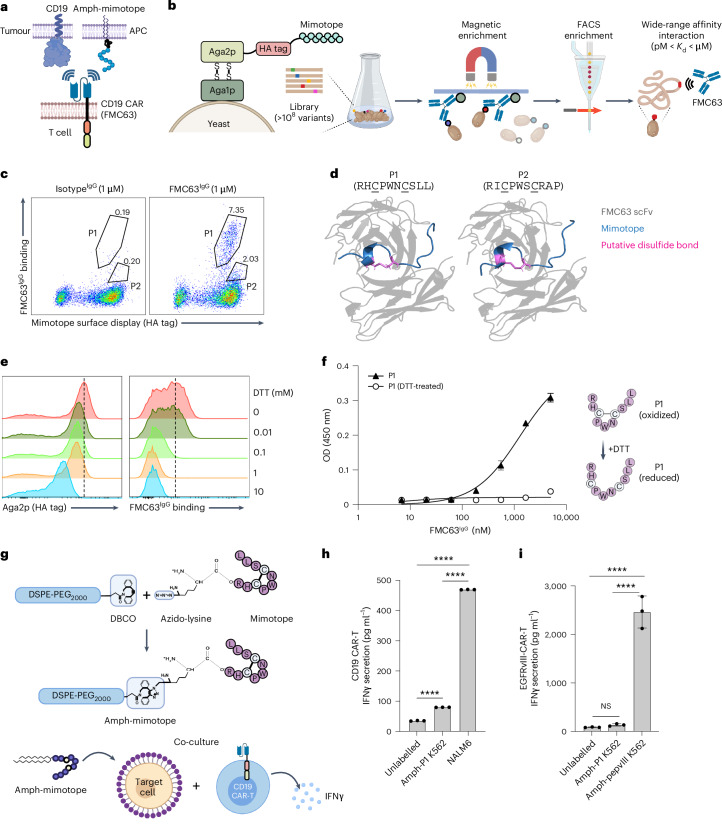
Yeast surface display identifies CD19 CAR binding mimotopes. **a**, Illustration of CD19 CAR stimulation by natural CD19 or amph-mimotope. Created in BioRender. Ma, L. (2025) https://BioRender.com/miyfij7. **b**, Yeast surface display workflow for identifying antibody-specific mimotopes. Created in BioRender. Ma, L. (2025) https://BioRender.com/5ugsnwl. **c**, Flow cytometry plots showing the successful identification of yeast cell populations (P1 and P2) binding to 1 µM FMC63^IgG^ after a single round of magnetic enrichment. **d**, Structural modelling of mimotopes P1 and P2 binding to FMC63. **e**, Effect of the disulfide bond on mimotope binding to FMC63^IgG^ on the yeast surface. Yeast cells bearing mimotope P1 were treated with increasing concentrations of DTT and then stained with 500 nM biotin-FMC63^IgG^ and an anti-HA tag antibody for analysis of binding by flow cytometry. **f**, ELISA analysis showing the impact of the disulfide bond on synthetic mimotope binding to FMC63^IgG^. Created in BioRender. Ma, L. (2025) https://BioRender.com/xd8q66t. **g**, Scheme for amph-mimotope generation by click chemistry linkage of azide-modified P1 to DBCO-PEG-DSPE, and schematic illustration of amph-P1 coating of target cells. Created in BioRender. Ma, L. (2025) https://BioRender.com/5ugsnwl. **h**, CD19^−^ K562 cells labelled with 100 nM amph-P1, unlabelled K562 cells or CD19^+^ NALM6 cells were co-cultured with CD19 CAR-T at a 5:1 E:T ratio for 6 h followed by IFNγ ELISA (****P < 0.0001). **i**, K562 cells unlabelled, labelled with 100 nM amph-P1 or labelled with 100 nM amph-pepvIII were co-cultured with control EGFRvIII-CAR-T cells at a 5:1 E:T ratio for 6 h followed by IFNγ ELISA. Error bars show mean ± s.d. with triplicate samples. ****P < 0.0001 by one-way ANOVA with Tukey’s post-test. Source data

We previously demonstrated that a CAR-specific amph-vaccine could provide potent stimulation of CAR-T cells in vivo^14,15^. The vaccine was created by linking a CAR ligand to an albumin-binding PEG-lipid, which upon co-administration with adjuvant would efficiently traffic to dLNs and decorate the surface of APCs, allowing presentation of the ligand to CAR-T cells in the lymph nodes (LNs) (Extended Data Fig. 1). We demonstrated this proof of concept using CARs that recognized arbitrary small molecule ligands (for example, fluorescein (FITC)) or linear peptide epitopes^14^. However, the antibody-based antigen-binding domain of the FDA-approved CD19 CARs (which use an antibody clone known as FMC63) binds to a conformational epitope spanning multiple domains of the human CD19 antigen^26^. Further, the CD19 extracellular domain is difficult to express and prone to misfolding^27^, making it challenging to use recombinant CD19 for manufacturing an amphiphile vaccine to stimulate CD19 CAR-T cells. As it is common for CAR binding domains to recognize complex conformational epitopes^28–30^, we sought to establish a general strategy to generate simple surrogate ligands for any CAR. We hypothesized that the generation of mimotopes, linear synthetic peptides that are also recognized by the CAR binding domain in addition to its native target antigen, could be an ideal option (Fig. 2a).

To identify mimotopes for FMC63, we used a yeast surface display library presenting ~5 × 10^8^ randomized linear peptides of 10 amino acids (AA, 10 mer) for screening against recombinant FMC63 expressed as a full-length IgG (hereafter FMC63^IgG^; Fig. 2b). Initial screening was performed using biotinylated FMC63^IgG^ attached to streptavidin beads for magnetic enrichment of weakly bound yeast clones. After a single round of screening using this enrichment protocol, flow cytometry analysis identified two yeast populations P1 and P2, which bound with higher or lower relative affinity to FMC63^IgG^, respectively, but not an isotype control antibody (Fig. 2c). The two peptides shared a common enriched sequence motif RXCPWXCXXX. The presence of two cysteines in the mimotope motif suggested the possibility that these mimotopes formed an intramolecular disulfide bond, with the proline between the two cysteines likely promoting a hairpin formation in the peptide structure^31^. Computational modelling of P1 (RHCPWNCSLL) and P2 (RICPWSCRAP) interactions with FMC63 using AlphaFold predicted that the mimotopes take on a curved alpha-helical structure, suggesting the formation of a cyclic structure with an intra-peptide disulfide bond (Fig. 2d).

To confirm the presence of a disulfide bond within the mimotope and test whether the disulfide is necessary for peptide binding to FMC63^IgG^, we treated the yeast clone P1 with increasing concentrations of the reducing agent dithiothreitol (DTT; Fig. 2e). Treatment with 10 mM DTT lowered Aga2p levels on the yeast cells by ~10-fold, likely owing to cleavage of the disulfide linkages anchoring the protein to the yeast cell surface. However, mild reductive conditions (that is, 0.1 mM, 1 mM DTT) largely preserved Aga2p on the yeast surface but eliminated FMC63^IgG^ binding (Fig. 2e). To further verify that this loss in antibody binding reflected loss of a disulfide in the mimotope itself, we chemically synthesized a cyclic version of mimotope P1 and monitored its binding to FMC63^IgG^ using an enzyme-linked immunosorbent assay (ELISA) in the presence or absence of pre-treatment of the peptide with DTT, and found that FMC63^IgG^ recognized plate-bound P1 with an apparent dissociation constant (*K*_d_) of 1.4 μM, but this binding was completely ablated for DTT-treated P1 (Fig. 2f). Next, we created amph-mimotope molecules by conjugating P1 functionalized with an N-terminal azide to DSPE-PEG_2__000_-DBCO through click chemistry (Fig. 2g). To test whether the amph-mimotope could insert in cell membranes and enable CD19 CAR-T cell recognition, we labelled CD19-negative K562 cells with amph-P1 and then incubated them with human CD19 CAR-T cells in vitro (Fig. 2g). Amph-P1-decorated K562 cells triggered detectable interferon-γ (IFNγ) secretion from CD19 CAR-T cells but to a much lesser extent than NALM6 cells (Fig. 2h). By contrast, amph-P1 failed to stimulate control EGFRvIII CAR-T cells, which responded robustly to amph-pepvIII (a minimal epitope from EGFRvIII; Fig. 2i). These results confirmed that P1 is a functional mimotope with a cyclic conformation required for its specific recognition by FMC63.

**Extended Data Fig. 4:**
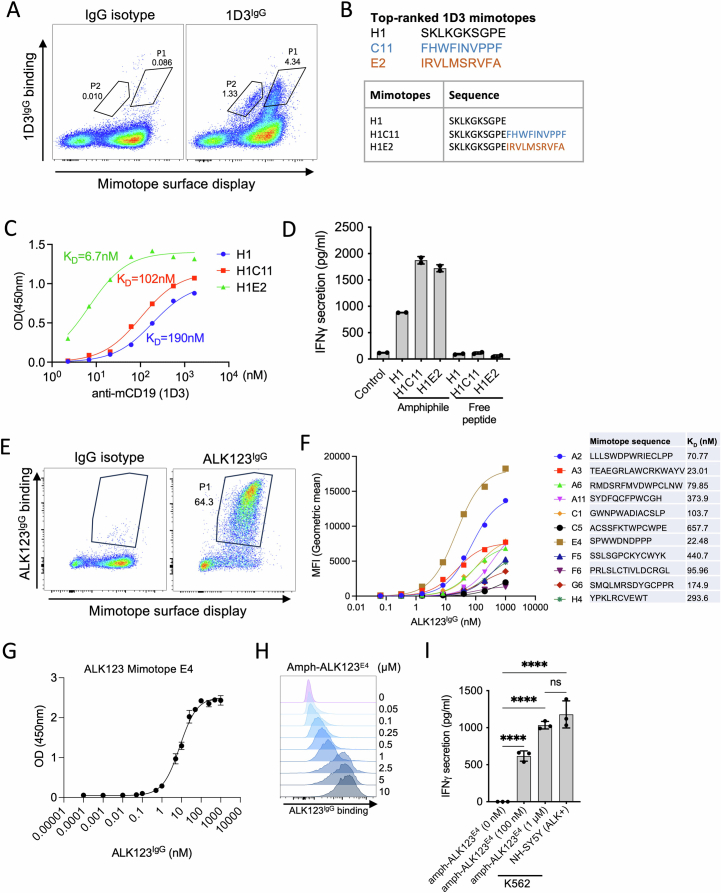
Identification of mimotopes specific for anti-mouse CD19 and anti-ALK antibodies. (**a-d**) Identification of a mimotope for anti-mouse CD19 mAb 1D3. (**a**) Flow cytometry scatter plots illustrating successful identification of yeast cells binding to 1D3^IgG^ at 1 μM. Two yeast populations, P1 and P2, were identified. (**b**) Top-ranked 1D3 mimotopes. Shown in the table are individual mimotopes and mimotope fusions. (**c**) ELISA showing the binding of 1D3^IgG^ to chemically synthesized mimotopes in B. A one site-specific binding model was used for assessing the apparent binding affinity. (**d**) Mouse IFN-γ secretion from anti-mouse CD19 CAR-T cells co-cultured with target cells labeled with 100 nM of amph-mimotopes or free mimotope peptides. Error bars show mean ± s.d. with three replicates. **, p < 0.01 by one-way ANOVA with Tukey’s post-test. (**e-f**) Identification of a mimotope for ALK-specific mAb ALK123. (**e**) Flow cytometry plots showing the successful identification of yeast cells binding to ALK123^IgG^ at 500 nM. (**f**) Binding of ALK123^IgG^ to select yeast clones. Amino acid sequences were shown for each yeast clone. A one site-specific binding model was used for assessing the apparent binding affinity. (**g**) ELISA showing the binding of ALK123^IgG^ to chemically synthesized ALK123 mimotope E4 (ALK123^E4^) (n = 3). Error bars show mean ± s.d. with three replicates (**h**) Representative histogram showing ALK123^IgG^ binding to target cells labeled with amph-ALK123^E4^. (**i**) Mouse IFN-γ secretion from anti-mouse ALK123 CAR-T cells co-cultured with target cells labeled with 100 nM or 1 μM of amph-ALK123^E4^ or NH-SY5Y (ALK^+^) cells (****, p < 0.0001). Error bars show mean ± s.d. with three replicates. ***, p < 0.001; ****, p < 0.0001; ns, non-significant by one-way ANOVA with Turkey’s post-test. Source data

To demonstrate that the yeast mimotope library is suitable for identifying mimotopes for any desired antibody, we repeated the library screen using an anti-mouse CD19 antibody (clone 1D3) (Extended Data Fig. 4a–d) and an anti-human ALK antibody of interest for CAR-T cell treatment of neuroblastoma^32–35^ (clone ALK123; Extended Data Fig. 4e–i). These additional library screens successfully identified multiple binders for each antibody. We chemically synthesized selected mimotopes for the ALK123 antibody and confirmed that the amph-mimotopes could effectively activate the corresponding CAR-T cells (Extended Data Fig. 4g–i), highlighting the general utility of yeast surface display to effectively identify antibody-specific mimotopes.

### Affinity maturation of the CD19 mimotope

**Fig. 3:**
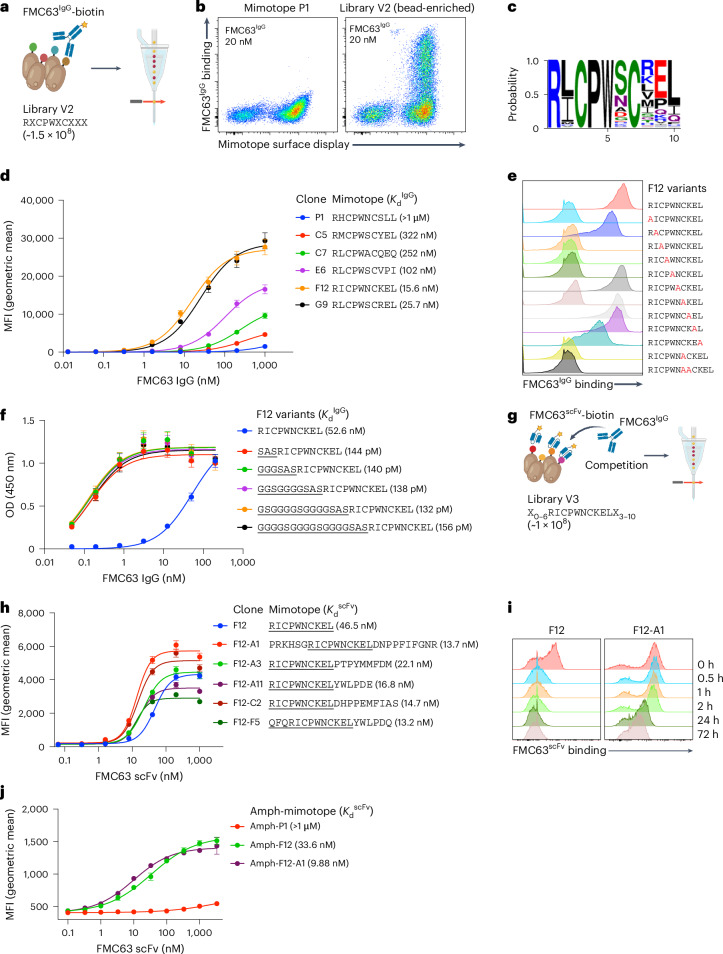
Affinity maturation of CD19 mimotopes. **a**, Schematic workflow for identifying affinity-enhanced mimotope variants of P1 from a motif-fixed mimotope library V2. Created in BioRender. Ma, L. (2025) https://BioRender.com/xd8q66t. **b**, Flow cytometry plots of 20 nM FMC63^IgG^ binding to yeast cells expressing the V2 mimotope library. The yeast clone P1 was included as a control. The V2 mimotope library was pre-enriched using FMC63^IgG^-coated magnetic beads (Methods). **c**, Weblogo showing the consensus mimotope sequence with high-affinity binding to FMC63^IgG^. **d**, Binding of FMC63^IgG^ to select yeast clones assessed by flow cytometry. Amino acid sequences and apparent binding affinity are shown for each yeast clone. Median fluorescence intensities (MFI) at each concentration were fit for the calculation of apparent *K*_d_ values. **e**, Impact of individual AA positions on FMC63^IgG^ binding. Yeast clones bearing F12 variants with 1-2 alanine (red) insertions or individual AA mutated to alanine (red) were assessed for FMC63^IgG^ binding by flow cytometry. **f**, ELISA showing the effect of flanking residues on mimotope^F12^ binding to FMC63^IgG^. The apparent binding affinity was determined as in **d**. **g**, Schematic workflow for identifying affinity-enhanced mimotopes from mimotope library V3 using kinetic sorting. Yeast library V3 was first stained with monovalent biotin-FMC63scFv, followed by competition with non-biotinylated bivalent FMC63^IgG^. Created in BioRender. Ma, L. (2025) https://BioRender.com/xd8q66t. **h**, Binding of FMC63^scFv^ to select yeast clones bearing various evolved mimotopes. The parental mimotope^F12^ sequence was underlined. The apparent binding affinity was determined as in **d** and specified after the mimotope sequence. **i**, Representative histograms showing retention of pre-bound FMC63^scFv^ on yeast clone F12-A1 in the presence of FMC63^IgG^. Yeast cells were pre-stained with 50 nM biotin-FMC63^scFv^ followed by incubation with 1 μM FMC63^IgG^. Yeast cells were sampled at indicated times for PE-streptavidin staining as an indicator for the retention of biotin-FMC63^scFv^. **j**, CD19^−^ K562 cells were labelled by incubation with 500 nM of the indicated amph-mimotopes, washed and then incubated with various concentrations of fluorescent FMC63^scFv^ followed by flow cytometry analysis of scFv binding; apparent binding affinities were determined as in **h**. Data are mean ± s.d. with triplicate samples. Source data

**Extended Data Fig. 5:**
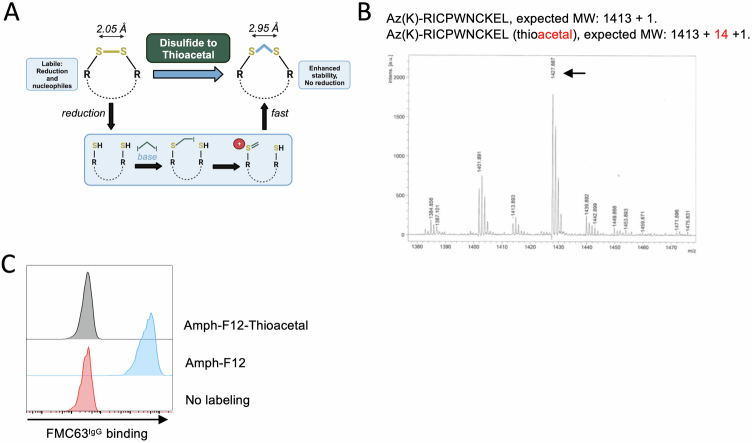
Converting the disulfide bond to a thioacetal bond prevents mimotope binding to FMC63^IgG^. **a**) schematics showing the chemical structure and conversion of the disulfide bond to a thioacetal bond. Created in BioRender. Ma, L. (2025) https://BioRender.com/xd8q66t (**b**) MALDI spectrum showing the purification of expected mimotope with a thioacetal bond. (**c**) Representative histogram showing FMC63^IgG^ binding to target cells labeled with amph-mimotope^F12^ variants.

The affinity of a typical CAR towards its ligand is often in the low nanomolar range^36^; however, mimotope P1 only exhibited a low micromolar apparent affinity towards FMC63^IgG^, and this apparent *K*_d_ is likely a substantial overestimate of the monovalent affinity of the peptide for FMC63 given the bivalent IgG format used in our binding assays. We therefore aimed to further evolve the mimotope to obtain variants with enhanced affinity. To this end, a mimotope library V2 was generated bearing a shared motif derived from the two parental mimotopes, namely RXCPWXCXXX, with a diversity of ~1.5 × 10^8^ (Fig. 3a). We pre-enriched the V2 mimotope library for binders using FMC63^IgG^-coated magnetic beads (Methods) and found that a substantial portion of this bead-enriched library V2 could be positively stained with 20 nM biotinylated FMC63^IgG^, while the original P1 yeast clone showed no detectable binding at this concentration (Fig. 3b). Flow cytometry sorting of the top 1% of the positive yeast cells followed by Sanger sequencing yielded dozens of mimotope clones. Sequence analysis of these mimotopes revealed a consensus sequence with high-affinity binding potential to FMC63^IgG^ (Fig. 3c). Several candidate mimotope clones resembling the consensus sequence were validated experimentally, and all of them exhibited stronger binding than the original clone P1 (Fig. 3d). We moved forward with mimotope F12 given its highest resemblance to the consensus sequence and highest binding affinity to FMC63^IgG^. This sequence was bound to FMC63 with an apparent *K*_d_^IgG^ of 15.6 nM (again, a value likely overestimating the true monovalent *K*_d_ owing to the avidity effect of the bivalent FMC63 IgG). Alanine scanning across all AA positions in the F12 peptide confirmed the dominant roles of R at position 1°, C at positions 3° and 7°, P at position 4° and W at position 5° (Fig. 3e). Residues at the remaining AA positions contributed more modestly to the overall affinity. Interestingly, inserting an extra 1-2 alanines between positions 6° and 7° completely disrupted F12 binding to FMC63^IgG^ (Fig. 3e), likely through the disruption of the intra-mimotope disulfide bond. Given the labile nature of the disulfide bond in the mimotope, we sought to test if the disulfide bond can be replaced with a structurally similar yet non-reducible thioacetal bond to produce a more stable mimotope (Extended Data Fig. 5a,b). Replacing the disulfide bond with a thioacetal bond completely eliminated its binding to FMC63^IgG^, perhaps because the thioacetal bond is 0.9 Å longer than the disulfide bond (Extended Data Fig. 5c).

Next, we sought to evaluate the contribution of the flanking sequence to mimotope binding with FMC63^IgG^. A 17 AA linker (GGGGS)_3_AS was initially introduced between the 10-mer mimotope and Aga2p during yeast library construction to avoid steric hindrance. We chemically synthesized F12 mimotope variants with increasing lengths of the upstream linker and performed ELISA to verify FMC63^IgG^ binding. Notably, the addition of even a minimal SAS tri-amino acid linker at the N terminus of the peptide increased binding of F12 to FMC63^IgG^ by a further ~100-fold (Fig. 3f). Given these data, we created a third yeast library V3 with ~1 × 10^8^ diversity with 0–6 and 3–10 flanking residues extended from the N and C termini of F12, respectively (Fig. 3g), aiming to identify mimotope variants with even higher affinity. We performed kinetic sorting by competing off pre-bound biotinylated monovalent FMC63^scFv^ using non-modified bivalent FMC63^IgG^ and were able to identify yeast populations with prolonged retention of biotinylated FMC63^scFv^, indicative of slower off-rates. Characterization of individual clones identified mimotope variants with further increased affinity compared with the parental F12 clone (Fig. 3h). Notably, clone F12-A1 exhibited a further ~10-fold increased affinity towards FMC63^scFv^ compared with F12. In the same competition assay as depicted in Fig. 3g, FMC63^scFv^ remained bound on the F12-A1 yeast surface over 24 h in the presence of excess soluble FMC63^IgG^ but was competed off the parental F12 clone within half an hour (Fig. 3i). Finally, we generated amphiphile conjugates of P1, F12 and F12-A1. When presented on the cell surface, these amph-mimotope variants exhibited apparent *K*_d_^scFv^ values of >1 μM, 33.6 nM and 9.9 nM, respectively (Fig. 3j). Therefore, these sequential affinity maturation screens effectively enabled the identification of mimotopes with a much-improved affinity for the CAR antibody.

### Mimotope does not interfere with FMC63 recognition of CD19

**Fig. 4:**
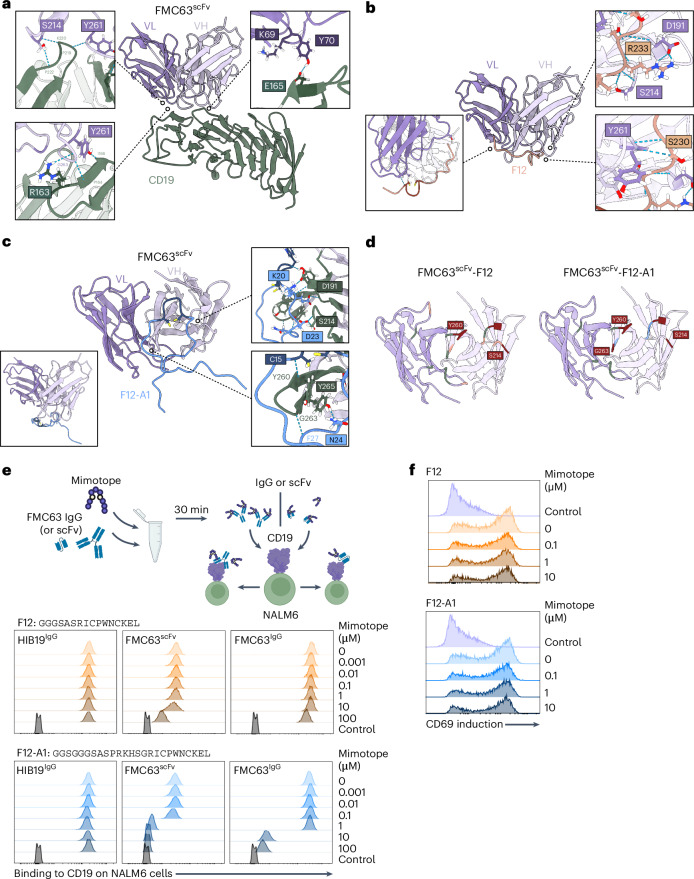
Structural modelling of mimotopes complexed with FMC63. **a**, Ribbon diagram of FMC63^scFv^ (purple) in complex with human CD19 (green, PDB 7URV). Interactions involving key residues of FMC63 are shown. Interacting side chain atoms are shown and boxed. Interactions involving backbone atoms are labelled without box. Created in BioRender. Ma, L. (2025) https://BioRender.com/yxmbsum. **b**,**c**, Computational models generated using AlphaFold and Rosetta for FMC63^scFv^ bound to F12 (**b**) (created in BioRender. Ma, L. (2025) https://BioRender.com/9lsos0m) or F12-A1 (**c**) (created in BioRender. Ma, L. (2025) https://BioRender.com/yxmbsum), respectively. Interactions involving previously mentioned key residues of FMC63 are labelled using the same scheme as in **a**. For F12-A1, the orientation of mimotope is shown to best exhibit how it engages FMC63. The panel in the bottom left depicts the complex in the same orientation as F12-FMC63. **d**, Interface footprint of mimotopes on FMC63. On the left side, the CD19 interface is shown in green, the F12 interface is shown in orange, and overlapping residues are shown in red. On the right side, the CD19 interface is shown in green, the F12A1 interface is shown in blue, and overlapping residues are shown in red (https://BioRender.com/yxmbsum). **e**, Recognition of natural CD19 recognition by FMC63^scFv^ in the presence of mimotopes. Biotinylated FMC63^scFv^ was pre-incubated with various concentrations of F12 or F12-A1 before staining NALM6 cells and detection via phycoerythrin (PE)-conjugated streptavidin. For FMC63^IgG^ binding, NALM6 cells were stained with the same concentrations of mimotope and FMC63^IgG^ with no previous incubation, and binding was detected using PE-conjugated anti-mouse IgG. A non-competing IgG clone HIB19 is shown as a control. Created in BioRender. Ma, L. (2025) https://BioRender.com/miyfij7. **f**, Activation of mimotope-bound CD19 CAR-T cells by target cells. CD19 CAR Jurkat T cells were pre-incubated with F12 or F12-A1 at indicated concentrations, and then co-cultured with NALM6 cells at a 1:1 E:T ratio for 16 h, followed by flow cytometry analysis of CD69 expression. Results in **e** and **f** are representative of three independent experiments.

To gain a comprehensive view of how the CD19 mimotope interacts with FMC63, we leveraged a recently solved crystal structure of FMC63^scFv^ in complex with CD19 (PDB 7URV)^28^ and modelled mimotope binding to FMC63^scFv^. Both F12 and F12-A1 were modelled using AlphaFold^37^ and Rosetta^38^, and the FMC63^scFv^-CD19, FMC63^scFv^-F12 and FMC63^scFv^-F12A1 binding interfaces were analysed using PDBePISA^39^. PDBePISA analysis recovered many of the previously defined interactions between FMC63^scFv^ and CD19 through analysis of polarities and bond lengths (Fig. 4a), confirming the validity of PDBePISA in performing interaction interface analysis. Structural modelling of F12 (Fig. 4b) or F12-A1 (Fig. 4c) interactions with FMC63^scFv^ showed that these mimotopes engage FMC63^scFv^ at the same domain. The key residues on FMC63^scFv^ that are essential for CD19 binding were found to be Y260, Y261, Y70, G263, W212, G262, S214, K69, Y265 and G129 (ref. ^28^). Of these key residues, both F12 and F12-A1 engage Y260 and S214 (Fig. 4d). However, only F12-A1 engages G263 (Fig. 4d), which likely explains its stronger binding to FMC63^scFv^ than F12.

To experimentally assess potential overlaps in the binding interface of FMC63 to CD19 versus our mimotope peptides, we pre-blocked FMC63^scFv^, FMC63^IgG^ or an alternative control anti-CD19 antibody HIB19^IgG^ with increasing concentrations of mimotopes F12 or F12-A1, and then added the antibodies to CD19^+^ NALM6 cells (Fig. 4e). When FMC63^scFv^ was pre-blocked with the F12 peptide, binding to NALM6 cells was only compromised when the F12 concentration was 100 µM, and NALM6 binding was nearly unaffected for FMC63^IgG^. By contrast, F12-A1 abolished FMC63^scFv^ or FMC63^IgG^ binding to CD19 when the mimotope concentrations were greater than 1 µM or 10 µM, respectively, consistent with its higher affinity for FMC63. However, when FMC63 CAR-expressing Jurkat T cells were pre-blocked with increasing concentrations of F12 or F12-A1 and stimulated with NALM6 cells, CD69 induction on Jurkat cells was not affected (Fig. 4f). Thus, despite some ability of the mimotopes to interfere with soluble FMC63 binding to CD19^+^ cells at high concentrations, FMC63-CAR-T cells retained their full potential of recognizing CD19^+^ leukaemic cells even in the presence of mimotopes.

### Amph-mimotope vaccination triggers CAR-T expansion in vivo

**Fig. 5:**
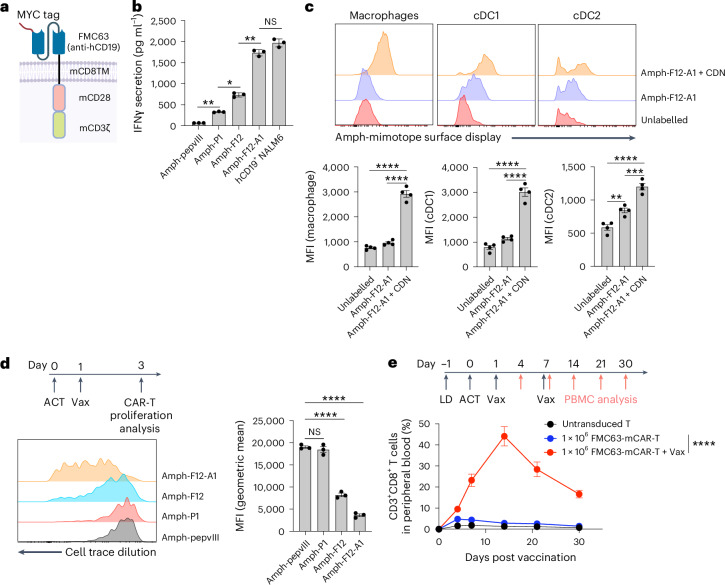
Amph-mimotopes require a threshold affinity to stimulate CAR-T proliferation in vivo. **a**, Schematic of hybrid FMC63-mCAR design. Created in BioRender. Ma, L. (2025) https://BioRender.com/xd8q66t. **b**, Comparison with various amph-mimotopes in stimulating FMC63-mCAR-T cells. K562 cells were labelled with 100 nM of amph-mimotope variants for 30 min, washed and co-cultured with FMC63-mCAR-T cells at a 1:1 E:T ratio for 6 h followed by ELISA measurement of IFNγ secretion (amph-pepvIII versus amph-P1 *P* = 0.0016; amph-P1 versus amph-F12 *P* = 0.0137; amph-F12 versus amph-F12-A1 *P* = 0.0090; amph-F12-A1 versus NALM6 *P* = 0.3338). **c**, Amph-mimotope labelling of APCs in vivo. C57BL/6 mice (*n* = 3 mice per group) were vaccinated with 10 nmol amph-mimotope ± CDG adjuvant, and 24 h or 48 h later, mimotope uptake by macrophages and cDCs was detected by staining with FMC63 followed by flow cytometry analysis. Shown are representative histograms of FMC63 staining (**P = 0.0055; ***P = 0.0006; ****P < 0.0001). **d**, Amph-mimotope vaccine stimulation of CAR-T cells in vivo. Wild-type C57BL/6 mice (*n* = 3 mice per group) received adoptively transferred with 2 × 10^6^ CTV-labelled FMC63-mCAR-T cells, and then vaccinated 1 day later with 10 nmol amph-peptides + 25 μg CDG adjuvant (Vax). Shown above is the timeline and below are representative histograms of FMC63-mCAR-T cell proliferation in LNs 48 h after vaccination. **e**, Longitudinal monitoring of CAR-T expansion in response to amph-mimotope vaccination. C57BL/6 mice (*n* = 5 mice per group) were lympho-depleted (LD), adoptively transferred with 10^6^ FMC63 CAR-T cells and then vaccinated at indicated time points, and circulating FMC63-mCAR-T cells were quantified in the blood by flow cytometry over time. Error bars show mean ± s.d. with triplicate samples for B, mean ± 95% CI for **c**–**e**. ***P < 0.0001; **P < 0.01, by one-way ANOVA with Tukey’s post-test for **b**–**d**, and two-way ANOVA with Tukey’s post-test for **e**. Source data

**Extended Data Fig. 6:**
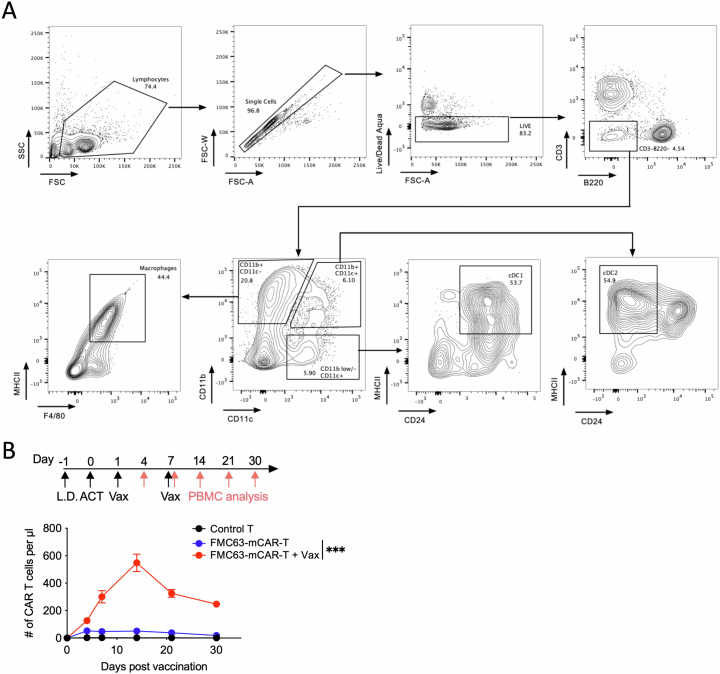
Amph-mimotope labeling of APCs and stimulation of FMC63-mCAR-T cell expansion *in vivo.* (**a**) Gating strategies and surface makers used for defining APC populations in the lymph node. (**b**) C57BL/6 mice (*n* = 5 animals/group) were lymphodepleted (L.D.) with 500 cGy gamma irradiation, adoptively transferred with 10^6^ FMC63-mCAR-T cells, and then vaccinated at indicated time points. Shown are the number of circulating FMC63-mCAR-T cells per microliter of blood quantified by flow cytometry over time. This data is related to Fig. 5e. Error bars show mean ± 95% CI. ***, p < 0.0001 by two-way ANOVA with Tukey’s post-test. Source data

To test if amph-mimotope vaccines could efficiently stimulate human CD19-targeting CAR-T cells in an immunocompetent mouse model in vivo, we established a hybrid CAR by fusing the FMC63 scFv to murine CAR signalling domains (FMC63-mCAR; Fig. 5a). First, we validated the ability of the amph-mimotope variants to stimulate FMC63-mCAR-T cells in vitro. K562 cells were pre-labelled with amph-mimotope variants and co-cultured with FMC63-mCAR-T cells as in Fig. 2g. Amph-mimotope-decorated target cells triggered IFNγ production from the CAR-T cells, with the stimulation efficiency positively correlating with FMC63-mimotope affinity and amph-F12-A1 providing the strongest CAR-T activation, comparable to stimulation by NALM6 cells (Fig. 5b). To determine if these amph-mimotopes could be effectively presented by DCs in vivo, we vaccinated C57BL/6 mice with 10 nmol amph-F12-A1, a dose that we previously found to be effective for amph-peptide stimulation of CAR-T cells. We formulated the mimotope with or without the cyclic dinucleotide (CDN) cyclic-di-GMP, a STING agonist added as an adjuvant^40^. Following vaccination, we isolated CD11b^+^ myeloid cells and CD11c^+^ DCs from the draining inguinal LNs and analysed the presence of surface-displayed amph-peptides using flow cytometry by staining the cells with biotinylated FMC63^IgG^ (Extended Data Fig. 6a). Interestingly, high levels of amph-mimotope were detected on APCs in dLNs for at least 24 h, but only when amph-mimotope was co-administered with CDN adjuvant (Fig. 5c).

Next, we labelled FMC63-mCAR-T cells with cell trace violet (CTV) and transferred them into C57BL/6 mice, followed by vaccination with amph-P1, amph-F12, amph-F12-A1 vaccine or saline as a control and flow cytometry analysis to detect CAR-T cell proliferation (CTV dilution) 2 days later. This functional test of amph-mimotope stimulation revealed a clear hierarchy of mimotope activity in vivo, with amph-P1 eliciting no CAR-T cell proliferation, amph-F12 eliciting limited proliferation and only amph-F12-A1 triggering proliferation of the entire CAR-T population (Fig. 5d). These data suggest that there is an affinity threshold for mimotope vaccines to effectively stimulate CAR-T cells in vivo.

Clinical evidence indicates that lack of antigen exposure soon after adoptive transfer may compromise CAR-T engraftment^41^. To test if amph-mimotope vaccine boosting post adoptive cell transfer could promote FMC63-mCAR-T cell expansion and engraftment in the absence of leukaemia, naive CD45.2^+^ C57BL/6 mice received sublethal lymphodepletion followed by intravenous (i.v.) infusion of either CD45.1^+^ FMC63-mCAR-T or control untransduced T cells (Fig. 5e). Without additional treatment, peripheral CD45.1^+^ CAR-T cells comprised <5% of total CD8^+^ T cells by day 4 and decreased to <3% by day 14 (Fig. 5e). By contrast, amph-F12-A1 vaccine boosting rapidly amplified CD45.1 CAR-T cells, reaching ~40% of total peripheral CD8^+^ T cells (~500 CAR-T cells per microlitre of blood; Fig. 5e and Extended Data Fig. 6b) by day 14 after two weekly vaccinations, and then slowly contracting over the next few weeks.

### Vaccine boosting of CAR-T in a mouse model of B-ALL/lymphoma

**Fig. 6:**
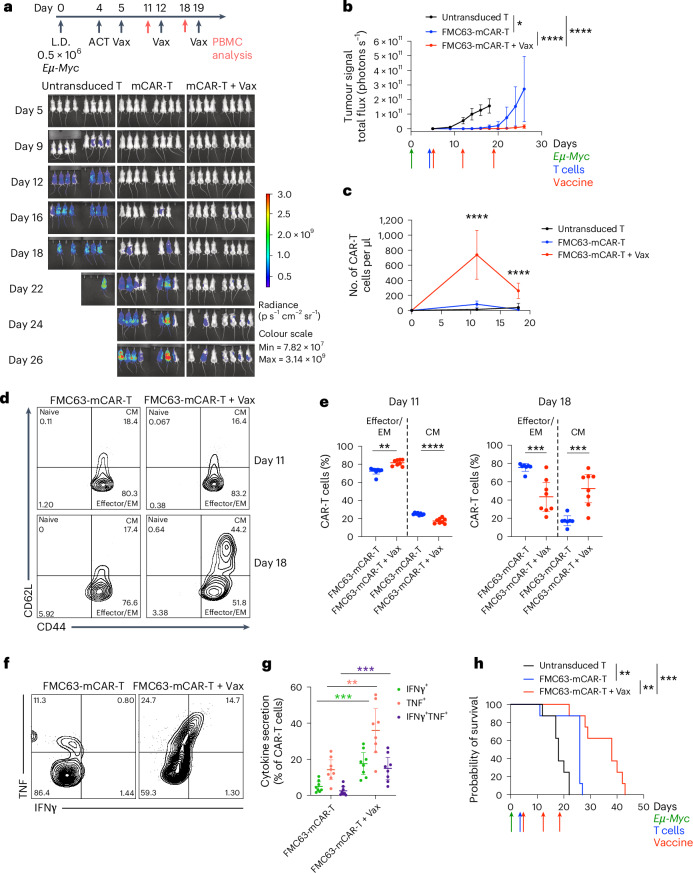
Amph-mimotope vaccine enhances FMC63-mCAR-T therapy in an immunocompetent hCD19^+^*Eμ-Myc* B-ALL/lymphoma mouse model. **a**,**b**, Real-time whole animal imaging tracking disease progression. C57BL/6 albino mice were lympho-depleted and injected with 0.5 × 10^6^ hCD19^+^Luc^+^ *Eμ-Myc* cells. On day 4, mice were adoptively transferred with 2 × 10^6^ FMC63-mCAR-T cells and then vaccinated 1 day later with 10 μg amph-F12-A1 + 25 μg CDG (*n* = 8 mice per group). Shown are whole animal imaging at indicated times (**a**) and quantification of total photon counts over time in each group (*P = 0.0100; ****P < 0.0001) (**b**). **c**, Enumeration of circulating CAR-T cells or control T cells by flow cytometry over time (*n* = 8 mice per group at days 0 and 11; for day 18 *n* = 5 for control T cells, *n* = 7 for FMC63-mCAR-T, *n* = 8 for FMC63-mCAR-T + Vax) (****P < 0.0001). **d**,**e**, Immunophenotype of circulating CAR-T cells on days 11 and 18. Shown are the representative flow cytometry plots (**d**) and percentages of effector/effector memory (EM; CD44^+^CD62L^−^) and central memory (CM; CD44^+^CD62L^+^) CAR-T cells (**e**) (*n* = 8 mice per group at day 11; *n* = 7 for FMC63-mCAR-T and *n* = 8 for FMC63-mCAR-T + Vax at day 11) (day 11 effector/EM **P = 0.0001; CM ****P < 0.0001; day 18 effector/EM ***P = 0.0007; CM ***P = 0.0004). **f**,**g**, Cytokine polyfunctionality of circulating CAR-T cells on day 11. Shown are representative contour plots (**f**) and percentages of cytokine-secreting CAR-T cells (**g**) (*n* = 8 mice per group) (IFNγ^+^, ***P = 0.0005; TNF^+^, **P = 0^.^0017; IFNγ^+^TNF^+^, ***P = 0.0006). **h**, The overall survival of *Eμ-Myc* mice under various treatment regimens (*n* = 8 mice per group) (untransduced T versus FMC63-mCAR-T, **P = 0.0015; untransduced T versus FMC63-mCAR-T + Vax, ***P = 0.0002; FMC63-mCAR-T versus FMC63-mCAR-T + Vax, **P = 0.0016). Error bars show mean ± 95% CI. *P = 0.0100; **P < 0.01; ***P < 0.001, ****P < 0.0001, by two-way ANOVA with Tukey’s post-test for **b**, by one-way ANOVA with Tukey’s post-test for **c**, by two-sided unpaired *t*-test for **e** and **g**, and by log-rank (Mantel–Cox) test for **h**. Source data

**Extended Data Fig. 7:**
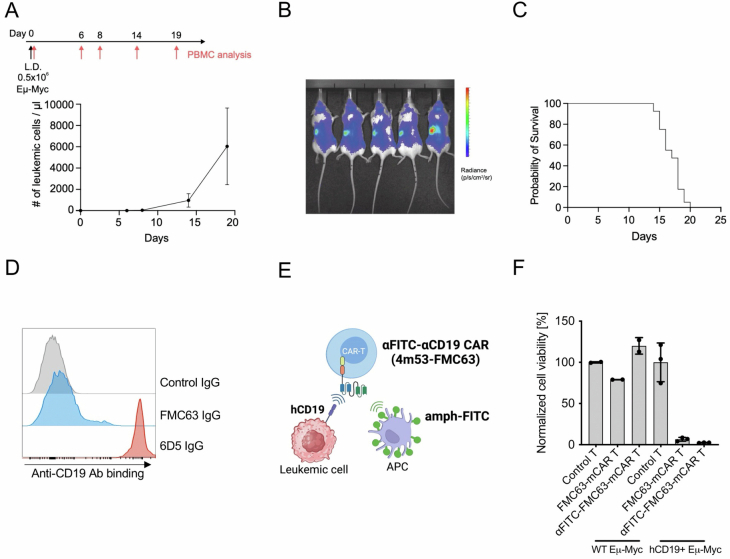
Establishment of the hCD19^+^ Eμ-Myc B-ALL/Lymphoma mouse model for assessing hCD19-targeted mCAR-T therapy. **a**) Experimental setup and timeline and enumeration of circulating leukemic cells (n = 4 animals). C57BL/6 albino mice were lymphodepleted (L.D.) and injected with 0.5x10^6^ hCD19^+^ Eμ-Myc cells. Peripheral blood was collected on days 0, 6, 8, 14 and 19. Created in BioRender. Ma, L. (2025) https://BioRender.com/xd8q66t (**b**) Representative IVIS imaging of Eμ-Myc albino mice at day 4. (**c**) Overall survival of untreated Eμ-Myc mice in this manuscript (n = 40). (**d**) FMC63 IgG binding to primary mouse B cells as monitored using flow cytometry. An anti-mouse CD19 IgG clone 6D5 was included as a control. (**e**) Schematics of tandem αFITC-FMC63-mCAR-T. (**f**) Cytotoxicity of FMC63 mCAR-T or tandem αFITC-FMC63-mCAR-T against WT (murine CD19^+^) or hCD19^+^ Eμ-Myc mouse B-ALL cells. A luciferase-based assay was used for assessing cell killing by CAR-T (see Methods). Error bars show mean±95% CI for A, mean ± s.d. (n = 2 for WT Eμ-Myc, n = 3 for hCD19+ Eμ-Myc) for F. Source data

**Extended Data Fig. 8:**
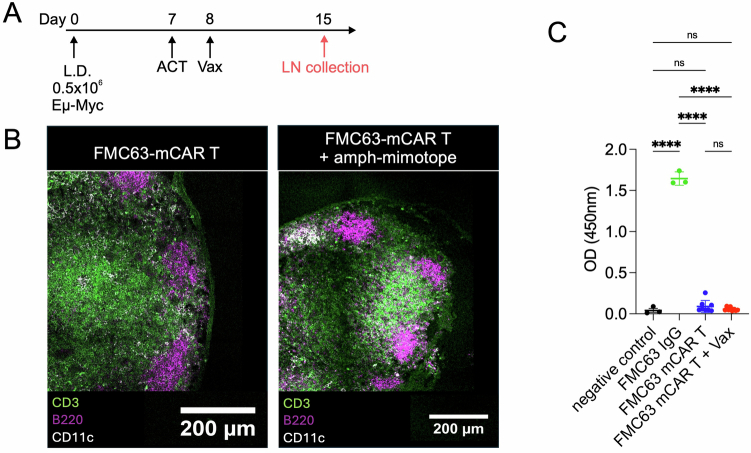
Evaluation of the impact of vaccine boosting of CAR-T cells on LN architecture and induction of serum antibodies against amph-mimotope vaccine. (**a**) Experimental setup and timeline. C57BL/6 mice were lymphodepleted (L.D.) and injected with 0.5x10^6^ hCD19^+^ Eμ-Myc cells. On day 7, mice were adoptively transferred with 2×10^6^ FMC63-mCAR-T cells or control T cells, then vaccinated 1 day later with 10 μg amph-F12-A1 (Vax). Inguinal (draining) lymph nodes were collected on day 15, frozen, and stained for CD3, B220, and CD11c. (**b**) Confocal imaging of LNs. Shown are LN sections stained with anti-CD3, anti-B220, and anti-CD11c to define the T cell zone, B cell zone and DC populations in the LN. (**c**) Evaluation of serum antibody against amph-mimotope vaccine. Serum was collected from FMC63-mCAR-T- and FMC63-mCAR-T + Vax-treated Eμ-Myc-bearing mice on day 21 (timeline presented in Fig. 6a) for analysis of mimotope-specific IgG by ELISA (n = 7 for FMC63-mCAR-T, n = 8 for FMC63-mCAR-T + Vax). FMC63 IgG was used as a positive control. This data is related to Fig. 6. Error bars show mean±95% CI. ****, p < 0.0001; ns, non-significant by one-way ANOVA with Tukey’s post-test. Source data

We next sought to evaluate effects of optimized amph-F12-A1 vaccine boosting of FMC63 CAR-T in an immunocompetent mouse model of CD19^+^ haematological malignancy. We transduced *Eμ-Myc* B-ALL/lymphoma cells^42^ with human CD19 and firefly luciferase. When these hCD19^+^Luc^+^ *Eμ-Myc* cells were inoculated into C57BL/6 mice, mice developed aggressive B-ALL/lymphoma with 100% penetrance and a median survival of 18 days (Extended Data Fig. 7a–c). FMC63^IgG^ showed minimal binding primary B cells expressing mouse CD19, and FMC63-mCAR-T effectively killed hCD19^+^ but not wild-type *Eμ-Myc* cells in vitro (Extended Data Fig. 7d–f). On day 4 after leukaemia engraftment, mice were infused with FMC63-mCAR-T alone or CAR-T combined with three weekly amph-mimotope vaccine boosts (Fig. 6a). Treatment with CAR-T alone resulted in moderate leukaemia control (Fig. 6a,b); however, CAR-T in combination with amph-mimotope boosting markedly suppressed disease progression (Fig. 6a,b). Enumeration of CAR-T cells in the blood confirmed that vaccine boosting elicited a rapid CAR-T expansion (Fig. 6c). Phenotypically, peripheral CAR-T cells from mice receiving vaccination were dominated by an effector population on day 11, but by day 18, nearly 50% of CAR-T cells in vaccinated mice exhibited a central memory phenotype compared with <20% in non-boosted mice (Fig. 6d,e). Vaccine-boosted CAR-T cells also possessed significantly higher cytokine polyfunctionality (Fig. 6f,g). As a result, mice receiving both CAR-T and amph-mimotope vaccine had greatly extended survival compared with those receiving CAR-T cells alone (Fig. 6h). LNs of amph-mimotope-boosted mice retained organized T cell and B cell areas, suggesting a lack of overt toxicity of the vaccine boost to dLNs (Extended Data Fig. 8a,b), and no mimotope-specific antibodies were induced (Extended Data Fig. 8c). Thus, amph-mimotope boosting of FMC63 CAR-T cells induced multiple favourable effects on CAR-T cell phenotype and increased the anti-tumour efficacy of CAR-T therapy.

**Extended Data Fig. 9:**
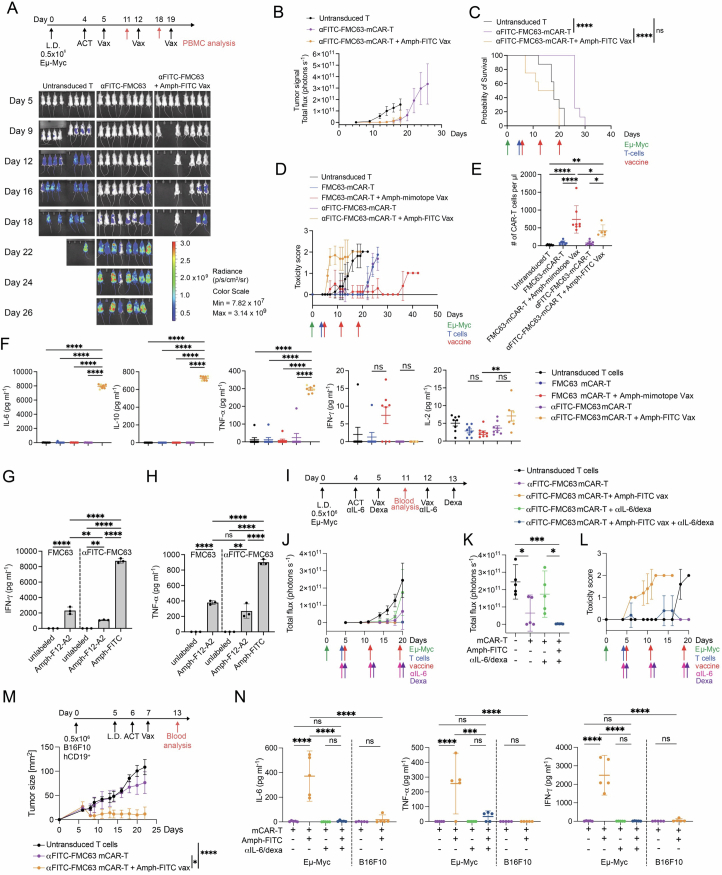
Toxicity of αFITC-FMC63-mCAR-T cells and amph-FITC vaccine therapy in a hCD19^+^ Eμ-Myc B-ALL/Lymphoma mouse model. (**a**) Experimental setup and whole animal imaging of disease progression. B6 albino mice were lymphodepleted (L.D.) and injected with 0.5x10^6^ hCD19^+^ Eμ-Myc cells. On day 4, mice were adoptively transferred with 2×10^6^ αFITC-FMC63-mCAR-T cells or FMC63-mCAR-T (mCAR-T), then vaccinated 1 day later with 10 nM amph-FITC (amph-FITC vax) or 10 μg amph-F12-A1 (amph-mimotope Vax) (n = 8 animals/group). Vaccinations were repeated 7 and 14 days later. mFMC63-MCAR-T, and FMC63-mCAR-T + Amph-mimotope Vax are the same as presented in Fig. 6. (**b**) Quantification of total photon counts over time in different treatment groups (n = 8 animals/group). (**c**) Eμ-Myc-injected mice were monitored for overall survival (****, p < 0.0001). (**d**) Toxicity score of each treatment group. Mice were monitored daily for clinical signs of toxicity before CAR-T injection and until mice reached the endpoint of the study. The toxicity of therapy was monitored using a toxicity score as described before^44^. A toxicity score of 0 corresponded to active, well-groomed animals, with well-kept hair coat and invisible spine. A score of 1 was assigned to mice with signs of hypomotility, tousled hair coat and a partially visible spine. A score of 2 was given in case of somnolence, rough, dull or soiled hair coat, hunched back with visible spine (n = 8 animals/group). (**e**) Enumeration of circulating CAR-T cells or control CD45.1^+^ T cells by flow cytometry on day 11. Control mice, mFMC63-MCAR-T, and FMC63-mCAR-T + Amph-mimotope Vax are the same as presented in Fig. 6c. (n = 8 animals/group; n = 5 for αFITC-FMC63-mCAR-T + Amph-FITC group). (FMC63-mCAR-T vs FMC63-mCAR-T + Amph-mimotope Vax, ****, p < 0.0001; Control T vs FMC63-mCAR-T + Amph-mimotope Vax, ****, p < 0.0001; αFITC-FMC63-mCAR-T vs αFITC-FMC63-mCAR-T + Amph-FITC Vax, *, p = 0.0316; FMC63-mCAR-T + Amph-mimotope Vax vs αFITC-FMC63-mCAR-T + Amph-FITC Vax, *, p = 0.0480; Control T vs αFITC-FMC63-mCAR-T + Amph-FITC Vax, **, p = 0.0088). (**f**) Serum cytokine analysis. Blood was collected on day 11 or day 7 for amph-FITC-treated mice (light yellow points), and a Cytometric Bead Array (CBA) was performed to determine the concentration of IL-6, IL-10, TNF-α, IFN-γ, and IL-2 in the serum (n = 8 animals/group; n = 7 for αFITC-FMC63-mCAR-T + Amph-FITC group) (****, p < 0.0001; **, p = 0.0057). (**g-h**) Cytokine responses of CAR-T cells to amph-vax labeled K562 cells. FMC63-mCAR-T and αFITC-FMC63-mCAR-T were co-cultured with K562 labeled with 500 nM Amph-F12-A2 or 500 nM Amph-FITC at a 10:1 E:T ratio for 6 hours followed by a measurement of IFN-γ and TNF-α concentration in supernatant by ELISA (n = 3). (**g**, ****, p < 0.0001; FMC63-mCAR-T + Amph-F12-A2 vs αFITC-FMC63-mCAR-T + Amph-F12-A2, **, p = 0.0017; αFITC-FMC63-mCAR-T + unlabeled vs αFITC-FMC63-mCAR-T + Amph-F12-A2, **, p = 0.0038. **h**, ****, p < 0.0001; αFITC-FMC63-mCAR-T + unlabeled vs αFITC-FMC63-mCAR-T + Amph-F12-A2, **, p = 0.0010). (**i**) Experimental setup and color coding used for **j-l**. B6 albino mice were lymphodepleted (L.D.) and injected with 0.5×10^6^ hCD19^+^ Eμ-Myc cells. On day 4, mice were adoptively transferred with 2×10^6^ αFITC-FMC63-mCAR-T cells +/- anti-IL-6 (αIL-6), then vaccinated 1 day later with 10 nM amph-FITC (amph-FITC vax) +/- dexamethasone (dexa). Vaccinations and αIL-6/dexamethasone treatment were repeated 7 and 14 days later (n = 5 animals/group). (**j**) Whole animal imaging of disease progression over time. (**k**) Total flux at day 20 (control T cells vs mCAR-T, *, p = 0.0118; mCAR-T + αIL-6/dexa vs mCAR-T + αIL-6/dexa + Vax, *, p = 0.0164; control T vs mCAR-T + αIL-6/dexa + Vax, ***, p = 0.0009). (**l**) Toxicity score of each treatment group. (**m**) Experimental setup and tumor size measurements. B6 albino mice were subcutaneously injected with 0.5×10^6^ hCD19^+^ B16-F10 cells. On day 5, mice were lymphodepleted (L.D.) followed by adoptive transfer of 2×10^6^ αFITC-FMC63-mCAR-T cells on day 6. On day 7, mice were vaccinated with 10 nM amph-FITC (amph-FITC vax). Vaccinations were repeated 7 and 14 days later (n = 5 animals/group). (**n**) Serum cytokine analysis. Blood was collected 6 days after vaccination, day 11 for Eμ-Myc-bearing mice or day 13 for B16F10-earing mice, and the CBA was performed to determine the concentration of IL-6, TNF-α, and IFN-γ in the serum (n = 5 animals/group) (****, p < 0.0001, ***, p = 0.0002). Error bars show mean±95% CI for B-F, J-N or mean±s.e.m. for G, H. *, p < 0.05, **, p < 0.01; ***, p < 0.001; ****, p < 0.0001; ns, non-significant by Long-rank (Mantel-Cox) test for C, one-way ANOVA with Tukey’s post-test for E-H, K, N; by two-way ANOVA with Tukey’s post-test for M. Source data

We previously reported amphiphile-fluorescein (amph-FITC) boosting of CAR-T cells bearing bivalent FITC/tumour antigen-specific CARs for solid tumours^14^. To compare amph-mimotope boosting of the FMC63-mCAR versus this alternative approach in the hCD19^+^ *Eµ-Myc* model, we synthesized a tandem FITC/hCD19-targeted murine CAR construct (that is, αFITC-FMC63-mCAR; Extended Data Fig. 7e), using the same high-affinity anti-FITC scFv that we previously tested^14^. In vitro, these dual-CAR-T cells effectively recognized and killed hCD19^+^ *Eµ*-*Myc* cells (Extended Data Fig. 7f). Treatment of *Eµ-Myc* tumour-bearing mice with tandem αFITC-FMC63-mCAR-T cells alone elicited similar tumour control as the monovalent FMC63-mCAR-T (Extended Data Fig. 9a–c; compare with Fig. 6a,b). However, while mice receiving treatment with the tandem CAR-T alone did not show signs of toxicity until the tumours progressed, all mice receiving the amph-FITC vaccine boost rapidly developed signs of severe toxicity immediately following the first vaccine boost, including hypothermia and hunched posture, and despite similar early tumour control to the non-boosted tandem CAR group, all mice receiving amph-FITC boosting died by day 20 (Extended Data Fig. 9a–d). By contrast, mice receiving FMC63-mCAR-T + the amph-mimotope vaccine showed no visible signs of toxicity during the treatment course (Extended Data Fig. 9d).

As the major difference between the amph-FITC and amph-mimotope vaccines is the affinity of the ligands for the CAR (the 4m5.3 scFv used in the FITC CAR binds to fluorescein with *K*_d_ of 270 fM)^43^, we hypothesized that these disparate outcomes reflect an over-stimulation of the tandem αFITC-FMC63-mCAR-T cells, leading to an exacerbation of cytokine release syndrome (CRS) when these cells encounter circulating tumour cells in the B-ALL/lymphoma model. Comparison of CAR-T cell levels in the blood showed that CAR-T expansion induced by the two vaccines was comparable, suggesting that increased toxicity following FITC boosting could not be attributed simply to the number of CAR-T cells present (Extended Data Fig. 9e). However, analysis of serum cytokines at day 6 after the first immunization (or earlier from moribund mice) showed severe CRS in mice receiving amph-FITC vaccine + tandem CAR-T as exemplified by massive elevations of serum interleukin-6 (IL-6), IL-10 and tumour necrosis factor (TNF) (Extended Data Fig. 9f). However, these signature CRS cytokines remained at low levels in mice receiving CAR-T only, or FMC63-mCAR-T plus amph-mimotope vaccination (Extended Data Fig. 9f). To assess if this discrepancy was triggered by the differential responses of CAR-T cells towards their respective vaccines, we co-cultured CAR-T cells with the amph-vax-labelled K562 cells in vitro and found that amph-FITC triggered much higher levels of IFNγ and TNF secretion by tandem CAR-T cells than the amph-mimotope elicited cytokine secretion from FMC63-mCAR-T cells (Extended Data Fig. 9g,h). In our previous work, we observed negligible toxicity of amph-FITC boosting of tandem CAR-T cells in solid tumour models^14^. Therefore, we performed a side-by-side comparison of the efficacy and toxicity of amph-FITC boosting of αFITC-FMC63-mCAR-T cells in C57BL/6 mice bearing either hCD19^+^ *Eµ-Myc* B-ALL/lymphoma (Extended Data Fig. 9i–l) or hCD19^+^ B16F10 melanoma (Extended Data Fig. 9m). While robust therapeutic effects were achieved in both cancer models (Extended Data Fig. 9j,m), CRS was only observed in *Eµ-Myc*-bearing mice (Extended Data Fig. 9n). Notably, anti-IL-6 and dexamethasone, a regimen used to manage CRS in CAR-T-treated patients, could alleviate the observed toxicities^44,45^. Indeed, anti-IL-6 and dexamethasone prevented the development of CRS and lethal toxicity without compromising the anti-leukaemic efficacy of amph-FITC-boosted αFITC-FMC63-mCAR-T cells (Extended Data Fig. 9j–l,n). These results suggest that in the setting of cancers where CAR-T cells encounter a substantial frequency of tumour cells in the blood, high-affinity ligand boosting can exacerbate CRS. This provides an important additional rationale for the mimotope discovery pipeline, where moderate-affinity ligands can be readily isolated for any given CAR.

### Mimotope-DC vaccine promotes leukaemia clearance by CAR-T

**Fig. 7:**
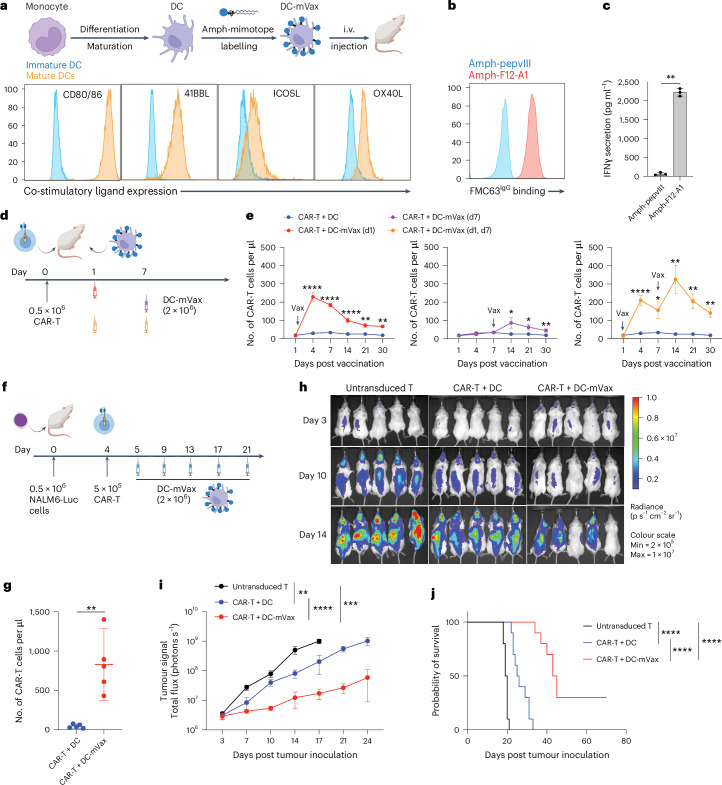
Amph-mimotope vaccine-labelled DCs augment human CD19 CAR-T therapy against leukaemia. **a**, Schematic of approach to generate DC-mVax and representative histograms depicting lipopolysaccharide/IFNγ-induced upregulation of co-stimulatory markers on mature DCs. Created in BioRender. Ma, L. (2025) https://BioRender.com/miyfij7. **b**, Representative histogram showing FMC63^IgG^ binding to DC-mVax. Activated monocyte-derived DCs were incubated with 500 nM amph-F12-A1 or control amph-pepvIII for 30 min, washed and then stained with FMC63^IgG^. **c**, CAR-T activation by DC-mVax. Activated monocyte-derived DCs were labelled with amph-F12-A1 or amph-pepvIII as in **b** and then co-cultured human CD19 CAR-T cells at a 1:1 E:T ratio. Shown is IFNγ measured in the supernatant after 6 h by ELISA (**P = 0.0013). **d**, Experimental set-up and timeline for **e**. Created in BioRender. Ma, L. (2025) https://BioRender.com/xd8q66t. **e**, Quantification of circulating human CD19 CAR-T cells in the absence or presence of a single dose of DC-mVax at day 1 or day 7 or two weekly doses of DC-mVax administered following timeline in **d** (*n* = 5 mice per group) (CAR-T + DC versus CAR-T + DC-mVax (d1), ****P < 0.0001; day 21, **P = 0.0030; day 30, **P = 0.0021; CAR-T + DC versus CAR-T + DC-mVax (d7), day 14, *P = 0.0468; day 21, *P = 0.0202; day 30, ***P = 0.0078; CAR-T + DC versus CAR-T + DC-mVax (d1, d7), ****P < 0.0001; *P < 0.0198; day 14, **P = 0.0057; day 21, **P = 0.0034; day 30, **P = 0.0027). **f**, Experimental set-up and timeline for **g**–**j**. Created in BioRender. Ma, L. (2025) https://BioRender.com/xd8q66t. **g**–**j**, Real-time whole animal imaging of B-ALL progression in NSG mice. Human CD19 CAR-T cells were administered with or without DC-mVax and tumour progression was monitored using in vivo bioluminescence imaging (*n* = 5 mice per group). Shown are the enumeration of circulating human CD19 CAR-T cells in peripheral blood on day 21 (**g**) (**P = 0.0079), whole animal imaging at indicated times (**h**) and quantification of total photon counts over time (**i**). Shown is one of two independent experiments (untransduced T versus CAR-T + DC, **P = 0.0011; untransduced T versus CAR-T + DC-mVax, ***P = 0.0004; CAR-T + DC versus CAR-T + DC-mVax, ****P < 0.0001). Overall survival of NSG mice under various treatment regimens (**j**). *n* = 10 mice assembled from two independent experiments. Shown are combined data from two independent experiments (****P < 0.0001). Error bars show mean ± s.d. with triplicate samples for **c**, and mean ± 95% CI for **e**, **g**, **i** and **j**. ***P < 0.0001; **P < 0.01 by paired two-sided Student’s *t*-test for **c**, two-sided Mann–Whitney test for **g**, by two-way ANOVA with Sidak’s post-test for **e**, by two-way ANOVA with Tukey’s post-test for **i** and by log-rank (Mantel–Cox) test for **j**. Source data

**Extended Data Fig. 10:**
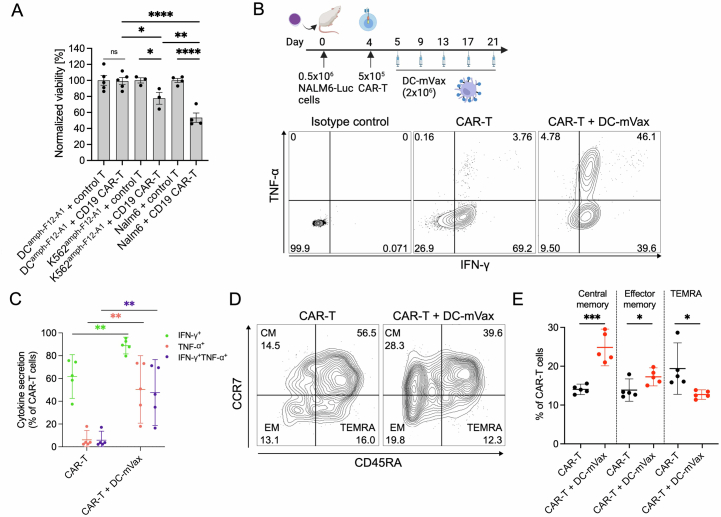
Resistance of amph-F12-A1-labeled DC to killing by CAR-T cells and immunophenotype of CAR-T cells boosted with DC-mVax. (**a**) Sensitivity of DC-mVax to CAR-T cells. Activated MoDCs or K562 cells were incubated with 500 nM amph-F12-A1 for 30 min, washed, and then co-cultured with human CD19 CAR-T cells or control T cells at a 10:1 E:T ratio for 6 hours followed by measurement of target cells viability by flow cytometry (n = 5 for MoDCs donors, n = 3 independent biological replicates for K562^amph-F12-A1^, n = 4 for CD19 CAR-T donors) (****, p < 0.0001; **, p = 0.0057; DC^amph-F12-A1^ + CD19 CAR-T vs K562^amph-F12-A1^ + CD19 CAR-T, *, p = 0.0200; K562^amph-F12-A1^ + control T vs K562^amph-F12-A1^ + CD19 CAR-T, *, p = 0.0241) (**b-c**) CAR-T cell cytokine polyfunctionality analysis. 0.5 x 10^6^ CAR-T cells were administered with or without DC-mVax as shown in the timeline (n = 5 animals/group). Spleens were harvested on day 19. Splenocytes were incubated for 6 hours with a cell stimulation cocktail followed by intracellular staining with anti-IFN-γ and anti-TNF-α antibodies. Shown are the representative contour plots (**b**, Created in BioRender. Ma, L. (2025) https://BioRender.com/xd8q66t) and the percentage of IFN-γ^+^, TNF-α ^+,^ and IFN-γ^+^TNF-α ^+^ CAR-T population (**c**) (IFN-γ^+^, **, p = 0.0063; TNF-α^+^, **, p = 0.0040; IFN-γ^+^TNF-α^+^, **, p = 0.0047). (**d-e**) Immunophenotyping of CAR-T cells in the spleen on day 19. Shown are representative contour plots (**d**) and the percentage of CAR-T (**e**) with CD45RA^−^CCR7^+^ central memory (CM), CD45RA^−^CCR7^−^ effector memory (EM), and CD45RA^+^CCR7^−^ effector memory re-expressing CD45RA (TEMRA) T cells phenotype (n = 5 animals/group) (Central memory, ***, p = 0.0003; Effector memory, *, p = 0.0337; TEMRA, *, p = 0.0246). Error bars show mean ± s.e.m. for A, or mean ± 95% CI. for C, E. *, p < 0.05; **, p < 0.01; ***, p < 0.001; ****, p < 0.0001; ns, non-significant by two-way ANOVA with Tukey’s post-test for A, by two-sided unpaired t-test for C, E. Source data

Human CAR-T cells are routinely evaluated in immunodeficient NSG mice, but this animal model is problematic for testing lymphatic-mediated amph-vaccine boosting, as these mice have generally defective LNs and lymphatic development owing to their lack of native lymphocytes^46^. To overcome this technical hurdle, we devised an alternative strategy to test amph-vaccine boosting of human CAR-T cells. We previously showed that amph-ligand vaccine molecules label LN-resident DCs, which are the key APC population mediating amph-vaccine stimulation^14^. To mimic this process in NSG mice in a human-relevant setting, we differentiated human peripheral blood monocytes into DCs in vitro and matured them with lipopolysaccharide and IFNγ (Fig. 7a). These matured human monocyte-derived DCs exhibited increased expression of multiple co-stimulatory molecules, including CD80/86, 41BBL, OX40L and ICOSL (Fig. 7a). Incubation of activated monocyte-derived DCs with amph-F12-A1 led to robust cell surface labelling with the mimotope (DC-mVax; Fig. 7b). DC-mVax efficiently stimulated human CD19 CAR-T cell activation in vitro (Fig. 7c) but were refractory to CAR-T cell killing (Extended Data Fig. 10a), consistent with our previous observations that DCs were not eliminated during vaccine boosting in vivo^14^, and work suggesting that DCs have mechanisms to resist CTL-mediated killing^47,48^. To determine whether DC-mVax could activate and expand human CD19 CAR-T cells in vivo in the absence of additional antigen, we infused human CD19 CAR-T cells into non-leukaemic NSG mice with or without subsequent i.v. infusion of DC-mVax (Fig. 7d,e). Mice receiving a single dose of DC-mVax 24 h after CAR-T infusion exhibited significant expansion of CAR-T cells in the peripheral blood compared with those receiving unmodified DCs, and circulating human CD19 CAR-T cells remained detectable at day 30 (Fig. 7e). Notably, the extent of CAR-T cell expansion markedly decreased when DC-mVax was infused 7 days after CAR-T cell transfer, indicating that early antigen exposure has an important role in promoting CAR-T expansion and engraftment (Fig. 7e). Repeated dosing of DC-mVax on days 1 and 7 led to a further increase in CAR-T cell expansion (Fig. 7e).

To determine whether DC-mVax boosting could augment the anti-tumour activity of human CD19 CAR-T cells, we established leukaemia in NSG mice by infusing luciferase-expressing NALM6 tumour cells as previously described^49^. Suboptimal doses of CD19 CAR-T cells were administered on day 4 followed 24 h later by repeated i.v. infusions of either unmodified DCs or DC-mVax (Fig. 7f). Despite the presence of high levels of target cells, CAR-T cells alone showed poor persistence/expansion compared with CAR-T cells boosted with DC-mVax (Fig. 7g). Notably, DC-mVax boosting significantly enhanced CAR-T cytokine polyfunctionality (Extended Data Fig. 10b,c) and differentiation towards a central memory phenotype (Extended Data Fig. 10d,e). While CAR-T cells alone only slightly delayed tumour expansion and moderately prolonged survival, the addition of the DC-mVax boosting enabled this dose of CAR-T cells to substantially slow tumour growth (Fig. 7h,i) and 30% of the mice completely rejected their tumours (Fig. 7j). Collectively, these results demonstrated that a suitably affinity-matured amph-mimotope could act as an effective vaccine to promote engraftment, expansion and reinvigoration of human T cells expressing the CAR currently used in all approved CD19-targeting CAR-T products and enhance their anti-leukaemic activity.

## Discussion

Successful engraftment and persistence is critical for ensuring robust and long-term anti-leukaemic activity by CAR-T cells^1,50^. Analysis of data from recent CAR-T cell clinical trials suggested that CD19-directed CAR-T cells initially expand more strongly if there is a high tumour burden, consistent with the idea of CAR-T expansion that is commensurate with the dose of antigen encountered. However, the long-term persistence of CAR-T cells does not correlate with the initial tumour burden, suggesting that this initial stimulus vanishes over time as leukaemic cells are cleared from the body and/or that stimulation received from tumour cells or naive B cells lacking appropriate co-stimulatory signals is insufficient to maintain the CAR-T cell population. We and others have recently demonstrated the ability of using a synthetic vaccine to boost CAR-T cells in vivo and achieved enhanced therapeutic outcomes^14,15,51^. Our strategy of linking a ligand for the CAR to an LN-targeting PEG-lipid amphiphile is simple but requires a suitable peptide or protein ligand. The poor expression and misfolding of recombinant human CD19 ectodomain^27^ make the use of the native target antigen challenging for this approach. Here we demonstrate that surrogate CAR ligands based on short peptides can be readily identified and affinity-matured using directed evolution, enabling the generation of mimotope vaccines that efficiently boost CD19 CAR-T cells analogous to natural CD19 antigen exposure. Vaccine boosting significantly impacted the outcome of CD19 CAR-T cell therapy by increasing CAR-T cell expansion and engraftment as well as augmenting CAR-T cell functionality. The mimotope vaccine discovered here is applicable to all four FDA-approved FMC63-based CD19 CAR-T cell therapies.

In addition to the human CD19 CAR mimotope, mimotopes for anti-mouse CD19 and anti-human ALK CARs were also successfully identified using the same yeast mimotope library, indicating the broad applicability of this library for identifying mimotopes for any desired antibody. Experimental verification and structural modelling demonstrated that the CD19 mimotope discovered through the yeast library forms a cyclic conformation that is essential for its recognition by FMC63. Given that FMC63 recognizes a conformational epitope, it is possible that a mimotope also needs to form a desired secondary structure to achieve strong binding. Notably, the CD19 mimotope only partially occupies the natural epitope binding domain on FMC63. This partial occupancy appears to enable FMC63-based CD19 CAR-T cells to maintain their capacity to recognize CD19^+^ leukaemia cells even in the presence of the mimotope, which could be important for allowing the CAR-T cells to continue sensing tumour cells in LNs during vaccine boosting. The transient surface presentation of amph-ligands also prevents CAR-T cells from receiving excessive and prolonged antigen exposure, thus reducing the likelihood of CAR-T cell exhaustion and severe CRS^14^.

We previously reported an alternative approach for vaccine boosting CAR-T, where the CAR itself is engineered as a tandem CAR with one binding domain recognizing a tumour antigen and the other domain binding to the synthetic molecule FITC, enabling boosting of these tandem CARs with the amph-FITC vaccine^14,15^. While this approach avoids the need to generate an amph-vaccine for each CAR product, many CARs (such as the FMC63-based CAR products) have already undergone extensive clinical testing, providing a strong motivation to leave the CAR design itself unchanged. In addition, the tendency of scFvs to undergo aggregation that can induce detrimental tonic signalling in CAR-T cells^52–54^ may make the bivalent CAR approach untenable for some CARs. In these settings, the amph-mimotope strategy is an ideal solution.

While B cells retain the antigen for CAR-T, evidence suggests that B cells themselves may not be as effective as conventional DCs for restimulating CAR-T cells at least partially owing to greater expression of co-stimulatory molecules in DCs^24^. Our previous findings showed that co-stimulatory molecules expressed by activated DCs are crucial for optimally expanding CAR-T cells with amph-ligands^14^. This DC-specific effect is further exemplified in our DC-mVax boosting of human CAR-T in NSG mice and amph-mimotope vaccine boosting of mouse CAR-T in the *Eμ-Myc* B-ALL/lymphoma mouse model, where vaccination not only drove CAR-T expansion but also enhanced effector cytokine expression and promoted memory differentiation. Conventional CD19 CAR-T cells expand and show efficacy following the first infusion; however, reinfusion of CAR-T cells into patients experiencing CD19^+^ tumour relapse is often ineffective, with significantly lower CAR-T expansion compared with the first infusion^41,55–57^. Therefore, one potential use of the mimotope vaccine is to support CAR-T cells during reinfusion. Notably, even during the first infusion, CD19 CAR-T cells rapidly decline after a transient expansion^9,58^. Despite the general correlation of initial CAR-T expansion with baseline tumour burden, a long-term follow-up study in B-ALL found that a higher ratio of peak CAR-T cell expansion to tumour burden significantly correlated with event-free survival and overall survival^21^. Although this effective ratio more likely occurs in patients with lower tumour burden, administering the mimotope vaccine upon CAR-T contraction during the first infusion could potentially reinvigorate CAR-T cells and sustain CAR-T expansion to increased this effective ratio in patients with both low and high tumour burden to improve event-free survival.

Given the toxicity associated with rapid CAR-T activation and expansion in patients with haematological malignancies, such as CRS and immune effector cell-associated neurotoxicity syndrome, amph-vaccine affinity, dose and dosing intervals may need to be tailored based on the CAR-T cell number and residual tumour burden in patients in future clinical trials. In our previous work, the amph-FITC vax/tandem CAR-T combination enhanced CAR-T therapy against solid tumours with transient mild toxicity^14^. However, in a side-by-side comparison, we found that the amph-FITC vax/tandem CAR-T combination triggered a lethal CRS in the immunocompetent *Eμ-Myc* B-ALL/lymphoma mouse model, while amph-mimotope vax/CAR-T treatment effectively controlled leukaemia progression with limited toxicity. Our data suggest that this drastic discrepancy in toxicity profiles is linked to the much higher affinity of the tandem CAR towards the amph-FITC vaccine versus the amph-mimotope binding to FMC63. Moreover, while target cells in solid tumours are typically confined to specific and localized areas, in haematological malignancies, target cells that provide signals for CAR-T activation are more abundant and widely distributed throughout the body, including the bloodstream, bone marrow and lymphatic system, where CAR-T cells preferentially traffic^59,60^. This is supported by a correlation of CAR-T cell expansion and initial tumour burden in CD19 CAR-T cell-treated patients as well as much higher expansion of CAR-T cells in patients with leukaemia/lymphoma compared with CAR-T-treated solid tumour patients^61^. These data indicate that amph-vaccine ligand affinity plays a critical role in the outcome of CAR-T boosting in cancers involving substantial levels of circulating tumour cells that will trigger cytokine secretion in the blood. The mimotope discovery pipeline described here is well suited to identify effective and safe ligands, as it allows mimotopes with a wide range of affinities to be discovered. CRS associated with vaccine-boosted CAR-T therapy, if present, can be effectively managed using anti-IL-6 and dexamethasone without compromising the therapeutic efficacy.

One set of challenges for characterizing amph-vaccines designed for human T cells are the deficiencies of current humanized mouse models. The defective lymphatics, absent or disorganized LNs, and poor crosstalk of mouse APCs with human T cells are barriers to evaluating vaccine boosting of human CAR-T cells in NSG mice^46^. As an alternative, we evaluated vaccine boosting of mouse T cells bearing a hybrid FMC63-mCAR in immunocompetent C57BL/6 mice via standard vaccine administration routes and boosting of human CD19 CAR-T cells using mimotope-decorated human monocyte-derived DCs in NSG mice via i.v. delivery. These two complementary approaches demonstrated the feasibility of the mimotope vaccine in the setting of natural trafficking and distribution from lymphatics to LNs, as well as its capacity to stimulate human CAR-T cells upon insertion into the surface of human DCs.

Messenger RNA vaccines encoding the target tumour antigen can also be used to stimulate CAR-T cells^51^. Recently, a CLDN6-expressing mRNA vaccine was tested in a phase 1 clinical trial in combination with CLDN6-directed CAR-T cells and showed promise in improving CAR-T cell expansion in patients with solid tumours^62^. CAR-T combined with mRNA vaccination was well tolerated, suggesting the overall safety profile associated with this combination therapy. However, mRNA–lipid nanoparticles differ from our amphiphile vaccines in their in vivo trafficking, biodistribution and the duration of antigen presentation on the cell surface. Therefore, their differential effects on boosting human CD19 CAR-T cells remain to be determined.

In summary, we have designed and validated a general strategy to generate mimotope ligands for any CAR. Using this methodology, we demonstrate an amph-mimotope vaccine that could be used clinically in tandem with all four FDA-approved CD19 CAR-T cell products. The ability of amph-mimotope vaccines to efficiently stimulate human CD19 CAR-T cells in vivo supports its potential use as a robust and clinically translatable approach for enhancing the engraftment, long-term persistence and anti-leukaemic activity of current CD19 CAR-T cell therapies.

## Methods

### Clinical trial data

#### Paediatric B-ALL

Patient data from two studies of tisagenlecleucel for the treatment of CD19-positive relapsed or refractory B-ALL in children and young adults, NCT01626495 (*n* = 60 infused)^18^ and NCT02906371 (*n* = 70 infused)^9^, were pooled (*n* = 130). In both studies, patients received lymphodepleting chemotherapy with fludarabine and cyclophosphamide followed by a pre-infusion bone marrow aspirate or biopsy to assess tumour burden, and infusion of tisagenlecleucel over 1–2 days. Patients were categorized based on the pre-infusion bone marrow aspirate or biopsy as high (≥40%, *n* = 40) or low tumour burden (<40%, *n* = 90). Peripherally circulating CD19 CAR-T cells were quantified by quantitative PCR (qPCR). Peripherally circulating normal B cells were quantified by flow cytometry. Data analyses were performed using R. The study protocols were approved by the Institutional Review Boards (IRBs) of the Children’s Hospital of Philadelphia and the University of Pennsylvania. Patients or their guardians provided written informed consent.

#### Adult B-ALL

We conducted a retrospective analysis of CAR-T expansion in a cohort of adult patients with B-ALL treated with the 4-1BB CART19 CTL019 (NCT02030847), now commercially known as tisagenlecleucel. Only patients with available pre-CART19 bone marrow involvement evaluation were included (*n* = 29). Patients’ characteristics were retrieved from the clinical trial database and electronic medical records as per an IRB-approved protocol. Tumour burden was defined as the percentage of bone marrow blast involvement before CAR-T infusion. Patients with more than 5% of blasts were defined as high tumour burden (*n* = 23), while patients with ≤5% blasts were considered low tumour burden (*n* = 6). Peak of CAR-T expansion was defined as the highest value of CAR19 copies per microgram DNA after CAR-T infusion, which typically occurs between days 7 and 14. CAR-T cell expansion in the peripheral blood was evaluated using qPCR at multiple time points, as previously described^19,18^. A Student’s *t*-test was used to compare CAR-T peak. Significance was set at 0.05.

#### Adult B cell lymphoma

We conducted a retrospective analysis of CAR-T expansion, according to tumour burden, in a cohort of patients with B cell lymphoma treated with 4-1BB CART19 CTL019 (NCT02030834), now commercially known as tisagenlecleucel. Only patients with LDH levels available at CART19 infusion were included (*n* = 37). Tumour burden was estimated per LDH serum levels at infusion day. Patients with LDH levels higher than 1.5-fold the upper normal limit were considered as high tumour burden (*n* = 7). Peak of CAR-T expansion was defined as the highest value of CAR19 copies per µg DNA after CAR-T infusion, which typically occurs between days 7 and 14. CAR-T cell expansion in the peripheral blood was evaluated using qPCR at multiple time points, as previously described^19,18^. Patients’ characteristics were retrieved from the clinical trial database and electronic medical records as per IRB-approved protocols. A *t*-test was used to compare CAR-T peak. Significance was set at 0.05.

### Cell line and animals

K562, Jurkat, NALM6 and 293 Phoenix cells were obtained from ATCC. The NALM6-Luc cell line was a gift from M. Birnbaum. The *Eμ-Myc* cell line was a gift from M. Hemann. Wild-type female C57BL/6 mice (CD45.2^+^), CD45.1^+^ congenic mice, B6(Cg)-*Tyr*^*c-2J*^/J (C57BL/6J albino) and NSG mice were purchased from the Jackson Laboratories. All animal studies were carried out following an IACUC-approved protocol following local, state and federal guidelines. The EBY100 yeast strain was a gift from K. D. Wittrup.

### Antibodies and recombinant proteins

FMC63 IgG, His-tagged FMC63 scFv, 1D3 IgG and anti-ALK IgG (ALK123) were cloned into the gWiz vector and used for recombinant protein production using Expi293 cells. Recombinant proteins were then purified using FPLC (Akta pure 25). Anti-hCD19 (HIB19) and anti-HA antibody were purchased from BioLegend. The anti-MYC antibody (9B11) was purchased from Cell Signaling.

### Yeast mimotope library construction

We followed previously established protocols^63,64^ to construct our yeast surface display libraries starting with the parental pCT-Con2 vector, except the following modifications that were tailored for the mimotope library. (1) The mimotope library PCR was carried out with nested PCRs. First PCR primers: mimotope Lib F, AACTAGCAAAGGCAGCCCCATAAACAC; mimotope Lib R, GATTTTGTTACATCTACACTGTTGTTATCAGATCTCGAGCTATTAMNNMNNM

NNMNNMNNMNNMNNMNNMNNMNNGCTAGCCGACCCTCCGCC. Second PCR primers: Min Cmyc EP F, GGCTCTGGTGGAGGCGGTAGCGGAGGCGGAGGGTCGGCTAGC; Min Cmyc EP R, GATTTTGTTACATCTACACTGTTGTTATCAGATCTCGAGCTATTA. PCR products were verified on a 2.5% agarose gel. The nested PCR products were extracted using the conventional pheno-chloroform and ethanol precipitation method, pelleted and dissolved in 15 μl H_2_O. The parental pCT-Con2 vector (Addgene number 41843) was digested with NheI, SalI and XhoI, purified using gel extraction followed by a clean-up step using PCR clean-up columns and eluted in 20 μl H_2_O. For electroporation, 4 μg digested backbone was pre-mixed with the entire PCR product with the maximum volume less than 20 μl. Then, pre-chilled yeast cells were added to the DNA mixture before transferring to an electroporation cuvette.

To generate mimotope library V2 with a fixed motif (RXCPWXCXXX), the mimotope Lib R primer was replaced with mimotope Lib V2 R, GATTTTGTTACATCTACACTGTTGTTATCAGATCTCGA

GCTATTAMNNMNNMNNACAMNNCCACGGACAMNNACG GCTAGCCGACCCTCCGCCTC. To generate mimotope library V3 with extended flanking sequences (X_0__–__6_RICPWNCKELX_3__–__10_), mimotope Lib R primer was replaced with

mimotope Lib V3 R1, GATTTTGTTACATCTACACTGTTGTTATCAGATCTCGAGCTATTA MNNMNNMNNAAGCTCCTTACAATTCCACGGACAAATACGMNNMNNMNN

GCTAGCCGACCCTCCGCCTC, to generate library X_3_RICPWNCKELX_3_;

mimotope Lib V3 R2, GATTTTGTTACATCTACACTGTTGTTATCAGATCTCGAGCTATTA AAGCTCCTTACAATTCCACGGACAAATACGMNNMNNMNNMNNMNNMNN

GCTAGCCGACCCTCCGCCTC, to generate library X_6_RICPWNCKEL;

mimotope Lib V3 R3,

GATTTTGTTACATCTACACTGTTGTTATCAGATCTCGAGCTATTA

MNNMNNMNNMNNMNNMNNAAGCTCCTTACAATTCCACGGACAAATACG

GCTAGCCGACCCTCCGCCTC, to generate library XRICPWNCKELX_6_;

mimotope Lib V3 R4, GATTTTGTTACATCTACACTGTTGTTATCAGATCTCGAGCTATTA

MNNMNNMNNAAGCTCCTTACAATTCCACGGACAAATACGMNNMNNMNNMNNMNNMNN

GCTAGCCGACCCTCCGCCTC, to generate library X_6_RICPWNCKELX_3_;

mimotope Lib V3 R5, GATTTTGTTACATCTACACTGTTGTTATCAGATCTCGAGCTATTA

MNNMNNMNNMNNMNNMNNAAGCTCCTTACAATTCCACGGACAAATACGMNNMNNMNN

GCTAGCCGACCCTCCGCCTC, to generate library X_3_RICPWNCKELX_6_;

mimotope Lib V3 R6, GATTTTGTTACATCTACACTGTTGTTATCAGATCTCGAGCTATTA

MNNMNNMNNMNNMNNMNNMNNMNNMNNMNNAAGCTCCTTACAATTCCACGGACAAATACGGCTAGCCGACCCTCCGCCTC, to generate library RICPWNCKELX_10_;

mimotope Lib V3 R7, GATTTTGTTACATCTACACTGTTGTTATCAGATCTCGAGCTATTA

AAGCTCCTTACAATTCCACGGACAAATACGMNNMNNMNNMNNMNNMNNMNNMNNMNNMNNGCTAGCCGACCCTCCGCCTC, to generate library X_10_RICPWNCKEL. A 10× diversity (definition of diversity, total number of mimotope variants within the yeast library) of all individual V3 libraries were mixed to create the final X_0__–__6_RICPWNCKELX_3__–__10_ library. Note that all the oligos were custom synthesized by Integrated DNA Technologies.

### Yeast surface display screen

To prepare biotinylated recombinant protein, 200 μl of antibody or scFv at 1 mg ml^−1^ in PBS was mixed with 10% volume of 1 M sodium bicarbonate to adjust pH to 7–8 followed by addition of EZ-Link sulfo-NHS-LC-biotin (Thermo Fisher) to reach a 5:1 biotin/protein molar ratio. The mixture was stirred at room temperature for 1 h, followed by spin-column purification and subsequent quantification using NanoDrop.

The yeast library was thawed, passaged and prepared as previously described^63,64^. To prepare for the screen, yeast cells were grown in SD-CAA until the OD600 reached 6–8. The 30× diversity (or at least 100 M) yeast cells were transferred into SG-CAA medium supplemented with 5% SD-CAA. Yeast were shaken at 20 °C overnight to induce protein display on the cell surfaces. We noticed that 5% of SD-CAA improved mimotope display on the yeast cell surface, and usually >75% induction could be achieved.

The yeast library screen was performed as previously described^63,64^ with the following modifications: (1) 15× diversity was used per sort (7.5 × 10^9^ for the mimotope library). Yeast was spun down at 3,500 RPM for 5 min, washed in cold 1× PBSA (1× PBS + 0.1% BSA) and then aliquoted into 2 × 2 ml tubes (3.75 × 10^9^ per tube). Yeast was then spun at 12,000*g* for 1 min and resuspended in 1 ml 1× PBSA. (2) For positive-sort beads (Dynabeads Biotin Binder (Thermo Fisher) pre-coated with 6.7–33 pmoles of biotinylated IgG or scFv as previously described^64^), non-bound yeast was gently removed, and the remaining beads were washed to remove unbound or weakly bound yeast cells with 1 ml 1× PBSA by inverting repeatedly. The supernatant was removed, and then yeast were resuspended in 1 ml SD-CAA, and counted to determine diversity. (3) 10× diversity of 1st round positive-sorted yeast was pelleted at 12,000*g* for 1 min. Yeast cells were inoculated into premade 5 ml SG-CAA with 5% SD-CAA and then induced at 20 °C overnight (at least 8 h is needed for reasonable induction). At least 20× diversity was pelleted and washed once with 1× PBSA, then resuspended in 1 ml 1× PBSA. (4) For the 2nd round of magnetic enrichment, the first bare bead sort and two subsequent negative sorts using beads coated with isotype control IgG were carried out at 0.5 beads per yeast cell for ≥2 h at 4 °C. This step was critical to remove background and non-specific binders. The final positive sort using FMC63_IgG_-coated beads was carried out following the standard enrichment procedure but with longer washes (30 s) to improve stringency. Enriched yeast cells were counted under microscopy to determine the diversity. At this point, the diversity should have been massively reduced. (5) 30× of enriched and induced yeast cells from the 2nd round of positive sorting were pelleted and stained with 30 μl 5 μM control IgG or FMC63 IgG at 4 °C for 30 min. Yeast was washed twice with 1 ml of 1× PBSA to remove residual antibody. When a plate was used for staining, yeast was washed 3–4 times with 200 μl. The pellet was stained in 50 μl of 1:100 dilution PE-streptavidin and BV421-HA for 20 min on ice. Yeast was washed twice with 1 ml of 1× PBSA before flow cytometry sorting (BD FACS Aria), with adjustments made as previously described for using a plate when needed. The top 0.5–1% of the major population based on FMC63 IgG binding was sorted. Usually following this modified protocol, a clearly distinct yeast population could be observed during flow cytometry analysis. (6) FMC63 IgG was used at 0.5 μM for the subsequent flow cytometry-based sort. We alternated between streptavidin and anti-biotin antibodies when staining yeast populations for flow cytometry to avoid selecting streptavidin binders. (7) For kinetic sorting, 10× of the library V3 was stained with 50 nM biotinylated FMC63 scFv for 30 min, washed 2× with 1× PBS and then incubated with 500 nM of non-modified FMC63 IgG for 1 h or overnight.

### Plasmid extraction from yeast cells and Sanger sequencing

The Zymo yeast DNA extraction kit was used with a modified protocol. In brief, 50 million yeast cells were pelleted and resuspended in 200 μl solution 1. Zymolyase (3 μl) was added, and then the mixture was vortexed and incubated at 37 °C for 1 h. Solution 2 (200 μl) was added and then mixed gently by flipping tubes upside down a few times and leaving at room temperature for 5 min. Solution 3 (400 μl) was added and then mixed well by shaking. The mixture was spun at top speed for 5 min. The supernatant was transferred to a new 1.5 ml EP tube and spun at top speed for 5 min to remove all white clumps. The clear lysate was transferred onto a spin column from a regular miniprep kit instead of the column provided in the Zymo yeast DNA extraction kit, and then spun at 11,000*g* for 30 s. Columns were washed with 600 μl of wash buffer from the miniprep kit, spun again at 11,000*g* for 1 min to dry the membrane, and then eluted with 50 μl of H_2_O. Eluted DNA was cleaned using a PCR clean-up kit (Invitrogen) following the manufacturer’s protocol. DNA was eluted in 20 μl H_2_O and quantified using NanoDrop. Clean DNA (5 μl) was transformed into 50 μl of DH5α commercial competent cells (NEB). The whole transformation was spread onto a 10 cm ampicillin agar plate. Individual bacteria colonies were picked and inoculated onto a 96-well agar plate and grown overnight. The entire plate was sent for miniprep and Sanger sequencing.

### ELISA

Mouse and human ELISA assays were performed following the manufacturer’s protocol (mouse and human IFNγ and TNF DuoSet, R&D Systems). For ELISA monitoring of FMC63 IgG binding to mimotopes, mimotope was dissolved in 1× PBS and coated onto a 96-well Maxisorp plate at 4 μg ml^−1^, 50 μl per well, sealed and kept at 25 °C overnight. The next day, the plate was washed 4× followed by 1 h blocking (1% BSA in 1× PBS) at 25 °C. After washing, FMC63 IgG was diluted to desired concentration in the blocking buffer and added to each well at 50 μl per well. After 1 h incubation at 25 °C, plates were washed again, the substrate was added, and the remainder of the assay was performed as previously described^14^. Anti-mimotope antibody response was determined by ELISA. Maxisorb plates (Thermo Fisher) were coated with 4 µg ml^−1^ of mimotope in PBS overnight at room temperature, washed 3× with 1× wash buffer (PBS containing 0.1% Tween-20 (v/v) and then blocked with blocking buffer (1% BSA in PBS) at 25 °C for 1 h. Serum (50 µl) diluted 1:100 was transferred into each well, covered and incubated for 1 h at 25 °C. Plates were then washed 3×, and 50 µl per well of goat anti-mouse IgG-HRP (BioRad, 1:5,000) was added and diluted in blocking buffer. Plates were incubated for 1 h at 25 °C and washed 3×, and 50 µl per well of TMB chromogen substrate solution (Thermo Fisher) was added. Plates were kept at 25 °C in the dark. After about 20 min, the reaction was terminated with 50 µl of 2 N H_2_SO_4_. Absorbances were read at 450 nm on a Tecan Spark plate reader (Tecan Life Sciences), with the subtraction of background reading at 540 nM. Mouse serum was collected on day 21.

### Construction of mouse and human CARs

Murine CAR-expressing constructs were generated by fusing geneblock fragments (custom ordered from IDT) into an MSCV retroviral vector. The hybrid FMC63-mCAR sequence is composed of a mouse CD8 signal peptide, FMC63 scFv, mouse CD8a hinge and transmembrane domain, CD28 co-stimulatory domain and CD3ζ intracellular domain as described in our previous work^14^. The tandem αFITC-FMC63-mCAR was constructed as described in our previous work^14^ by fusing an αFITC scFV (clone 4m5.3) to the N terminus of the FMC63-mCAR (CD28) via a (G4S)4 linker with the mouse CD8 signal peptide at the N terminus of the αFITC scFV, and a Myc tag was inserted immediately after the FMC63 scFv and before the mouse CD8 hinge to facilitate the detection of CAR expression on the T cell surface. The human CAR-expressing constructs were generated by fusing geneblock fragments (custom ordered from IDT) into a lentiviral vector containing the EF1a promoter. The CD19 CAR is composed of a human CD8 signal peptide, FMC63 scFv (VLVH), human CD8 hinge and transmembrane domain, CD28 co-stimulatory domain and CD3ζ intracellular domain as described previously^14^. To facilitate CAR detection by flow cytometry, a MYC tag was inserted at the N terminus of FMC63 scFv immediately following the signal peptide in both constructs. The mouse ALK123 CAR was designed by fusing an N-terminal MYC-tagged ALK123 scFv to the mouse CD8α hinge and transmembrane domain, followed by the murine CD28 co-stimulatory domain and CD3ζ signalling domain as described before^35^.

### Virus production

For optimal retrovirus production, 293 Phoenix cells were cultured till 80% confluence and then split at 1:2 for further expansion. Twenty-four hours later, 5.6 × 10^6^ cells were seeded in a 10 cm dish and cultured for 16 h till the confluency reached 70%. Thirty minutes to 1 h before transfection, each 10 cm dish was replenished with 10 ml pre-warmed medium. Transfection was carried out using the calcium phosphate method following the manufacturer’s protocol (Clontech). In brief, for each transfection, 18 μg of plasmid (16.2 μg of CAR plasmid plus 1.8 μg of Eco packaging plasmid) was added to 610 ml of ddH_2_O, followed by addition of 87 ml of 2 M CaCl_2_. Seven hundred millilitres of 2× HBS was then added in a dropwise manner with gentle vortexing. After a 10 min incubation at 25 °C, the transfection mixture was gently added to Phoenix cells. After 30 min incubation at 37 °C, the plate was checked for the formation of fine particles, as a sign of successful transfection. The next day, old medium was removed and replenished with 8 ml of pre-warmed medium without disturbing the cells. The virus-containing supernatant was collected 36 h later and passed through a 0.45 μm filter to remove cell debris, designated as the ‘24 h’ batch. Dishes were refilled with 10 ml of fresh medium and cultured for another 24 h to collect viruses again, designated as the ‘48 h’ batch, this process can be repeated for another 2 days to collect a ‘72 h’ batch and ‘96 h’ batch. All virus supernatant was aliquoted and stored at −80 °C. Virus transduction rate was evaluated in a 12-well format by mixing 0.5 million activated T cells with 0.5 ml of viruses from each batch. Plate coating, spin infection and flow cytometry analysis of CAR expression were carried out as described below. In the majority of experiments, the ‘48 h’ and ‘72 h’ batches yielded viruses that transduced T cells at 90–95% efficiency, and the ‘24 h’ and ‘96 h’ batch viruses led to >80% transduction. Only viruses with >80% transduction rate were used for animal studies. Lentivirus was produced as previously described^65^.

### Primary mouse T cell isolation and CAR-T cell production

For T cell activation, 6-well plates were pre-coated with 5 ml of anti-CD3 (0.5 μg ml^−1^, clone 2C11) and anti-CD28 (5 μg ml^−1^, clone 37.51) per well at 4 °C for 18 h. CD8^+^ T cells were isolated using a negative selection kit (Stem Cell Technology) and seeded onto pre-coated 6-well plates at 5 × 10^6^ cells per well in 5 ml of complete medium (RPMI + penicillin/streptomycin + 10% FBS + 1× NEAA + 1× sodium pyruvate + 1× 2-mercaptoethanol + 1× ITS (insulin-transferrin-swelenium, Thermo Fisher)). Cells were cultured at 37 °C for 48 h without disturbance. Twenty-four hours before transduction, non-TC-treated plates were coated with 15 μg ml^−1^ retronectin (Clontech). On day 2, cells were collected, counted and resuspended at 2 × 10^6^ cells per ml in complete medium supplemented with 20 μg ml^−1^ polybrene and 40 IU ml^−1^ mIL-2. Retronectin-coated plates were blocked with 0.05% FBS containing PBS for 30 min before use. The virus supernatant (1 ml) was first added into each well of the blocked Retronectin plate, and then 1 ml of the above cell suspension was added and mixed well by gentle shaking to reach the working concentration of polybrene at 10 μg ml^−1^ and mIL-2 at 20 IU ml^−1^. Spin infection was carried out at 2,000*g* for 120 min at 32 °C. Plates were then carefully transferred to an incubator and maintained overnight. On day 3, plates were briefly centrifuged at 1,000*g* for 1 min, and virus-containing supernatants were carefully removed. Fresh complete medium (3 ml) containing 20 IU ml^−1^ of mIL-2 was then added into each well. Cells were passaged 1:2 every 12 h with fresh complete medium containing 20 IU ml^−1^ of mIL-2. Transduction efficiency was evaluated by surface staining of a MYC tag included in the CAR construct^14^ using an anti-MYC antibody (Cell Signaling, clone 9B11) or by the GFP level for ALK123 CAR^35^ ~30 h after transduction. If needed, on day 3, after flow cytometry analysis of virus transduction, CAR-T cells could be frozen down and stored for assays at a later time. For in vivo experiments, CAR-T cells were used on day 4. For in vitro experiments, CAR-T cells were cultured until day 5.

### Primary human T cell isolation and CAR-T cell production

Buffy coats were obtained from anonymous healthy donors (Research Blood Components, LLC). Total peripheral blood mononuclear cells (PBMCs) were isolated by Ficoll-Paque PLUS gradient separation. CD8^+^ T cells were isolated directly using the EasySep human CD8^+^ T cell isolation kit (STEMCELL). For experiments completed at the Children’s Hospital of Philadelphia (CHOP), CD3^+^ T cells were isolated by negative selection using RosettaSep kits from STEMCELL Technologies and obtained from Human Immunology Core at the Perelman School of Medicine at the University of Pennsylvania. T cells were activated with Human T-Activator CD3/CD28 Dynabeads (Thermo Fisher) at a bead-to-cell ratio of 3:1 in complete medium supplemented with 30 IU ml^−1^ recombinant human IL-2 (PeproTech). After 2 days of activation, T cells were transduced with lentiviral supernatants as described above for murine T cells, and transduction efficiencies were determined by flow cytometry 2 days later. If transduction efficiency was less than 50%, CAR^+^ T cells were enriched by staining total expanded T cells with a PE-conjugated anti-MYC antibody followed by staining with anti-PE microbeads and magnetic selection for CAR^+^ T cells. Enriched CAR-T cells were continuously expanded till day 7 for adoptive transfer to NSG mice.

### Mimotope, amph-mimotope production and vaccination

Various mimotopes were custom synthesized (GenScript). The mimotope with a thioacetal bond was synthesized according to a previously published protocol^66^ and verified by MALDI. Amph-mimotope molecules were produced and purified as previously described with modifications as indicated below^14^. In brief, mimotope peptides (with an N-terminal azido lysine) were dissolved in H_2_O at 10 mg ml^−1^ and mixed with 1.1 molar equivalent of DSPE-PEG_2__000_-DBCO (Avanti). The mixture was agitated at 25 °C for 24 h. Conjugation efficiency was analysed using a C4 column on HPLC (Shimadzu). Typically, the conjugation efficiency reached >95%. When the conjugation efficiency was low, unconjugated peptide was removed using HPLC and the conjugates were collected. The resulting products were lyophilized, re-dissolved in PBS, quantified using NanoDrop and stored at −20 °C. DSPE-PEG-FITC was purchased from Avanti. For vaccination, unless otherwise stated, mice received weekly subcutaneous (s.c.) injection of 10 μg peptide equivalent of amph-mimotope mixed with 25 μg of cyclic-di-GMP (CDG; Invivogen) in 100 μl 1× PBS, administered 50 μl to each side at the tail base.

### In vitro and in vivo amph-mimotope labelling

For in vitro labelling, target cells were pelleted at 1,000*g* for 3 min and washed with PBS twice to remove residual protein. The cell pellet was then resuspended at 1 × 10^6^ cells per ml in PBS containing amph-mimotope molecules at the indicated concentrations and incubated at 37 °C for 30 min. The labelling reaction was stopped by pelleting cells and washing with PBS twice. For in vivo labelling, mice received 10 μg peptide equivalent of amph-mimotope with or without CDG as described above. Twenty-four hours later, inguinal LNs were extracted and dissociated into single cell suspension for flow cytometry staining for macrophages (MHCII^+^CD11b^+^CD11c^−^F4/80^+^), cDC1 (MHCII^+^CD11c^+^CD11b^low/−^CD24^+^) and cDC2 (MHCII^+^CD11c^+^CD11b^+^CD24^low/−^) as previously described^15^. To detect amph-mimotope decoration of various LN cell populations, 100 nM of biotinylated FMC63 IgG was included in the antibody cocktail followed by secondary staining with AlexaFluor 647-streptavidin. For amph-ALK123 mimotope E4, target cells were labelled as described before, stained with biotinylated 100 nM ALK123^IgG^ followed by secondary staining with PE-streptavidin.

### CAR-T functionality assay

The functionality of CAR-T cells was assessed by co-culturing with target cells in vitro. Ninety-six-well U bottom plates were used. Unless otherwise stated, 1 × 10^5^ sorted CAR-T cells or unsorted CAR-T cells (if >70% CAR^+^ T cells) possessing an equivalent number of CAR^+^ T cells were mixed with 1 × 10^4^ target cells in a total volume of 200 μl complete medium containing 20 IU ml^−1^ of mIL-2 (for mouse hybrid CAR-T) or 30 IU ml^−1^ of hIL-2 (for human CAR-T). After 6 h co-culture, cells were pelleted at 2,000*g* for 5 min, and supernatants were collected for IFNγ ELISA.

### Modelling mimotope interaction with FMC63

Both F12 and F12-A1 were modelled as multimers using AlphaFold with default settings^37^. AlphaFold generated 25 structures that were relaxed using the AMBER force field option, and then ranked internally. All 25 structures were clustered with Rosetta^38^ using a root mean square deviation (RMSD) cut-off of 2.0 Å to better understand which conformations were favoured during modelling. For both F12 and F12-A1, the top-ranked model resided in the cluster with the most members, and the most favourable energy when scored using the Rosetta score function^67,68^. The top-ranked structure was minimized using the Rosetta FastRelax^69^ protocol with deviations up to 3.0 Å allowed, to eliminate any clashes between side chains and achieve a more favourable conformation when scored using Ref2015. FastRelax produced 100 constructs, of which the construct with the most favourable energy was designated as the candidate structure. PDB 7URV was similarly minimized, with deviations up to 0.5 Å allowed, to ensure that coordinates are constrained to the crystal structure. All interfaces were analysed using the PDBePISA web server^39^.

### In vivo tracking of CAR-T proliferation and response to amph-mimotope stimulation in syngeneic mouse model

To monitor short-term amph-mimotope stimulation of CAR-T cells in vivo, 2 × 10^6^ murine hybrid FMC63-mCAR-T cells and untransduced T cells were mixed at a 1:1 ratio and labelled with 2.5 μM CTV and intravenously infused into recipient C57BL/6 mice. Sixteen hours later, 100 μl of amph-mimotope vaccine (10 nmol of amph-mimotope mixed with 25 μg CDG in 100 μl PBS) was s.c. injected into recipient mice, with 50 μl on each side of the tail base. After an additional 48 h, mice were euthanized and inguinal LNs were excised for flow cytometry analysis. Live CD3^+^CD8^+^CTV^+^ cells were gated as donor cells, and staining for the Myc tag on the CAR was used to distinguish CAR-T cells from non-CAR-T cells. For long-term monitoring of hybrid FMC63-mCAR-T cell expansion in response to amph-mimotope vaccination, recipient CD45.2 mice received sublethal lymphodepletion (500 cGy gamma irradiation) on day −1 followed by i.v. infusion of 1 × 10^6^ CD45.1 FMC63-mCAR-T cells on day 0. Two weekly doses of amph-mimotope vaccines were given on day 1 and day 7, and peripheral blood was sampled on day 4, day 7 and then every week thereafter for enumeration of CAR-T cells.

### Preparation of monocyte-derived DCs and amph-mimotope labelling

PBMCs were isolated from a buffy coat using Ficoll-Paque density gradient. CD14^+^ monocytes were then purified using the pan monocyte isolation kit (Miltenyi Biotec) or isolated by negative selection using RosettaSep kits from STEMCELL Technologies and obtained from Human Immunology Core at the Perelman School of Medicine at the University of Pennsylvania. The characterization and differentiation of monocyte to immature DCs were performed as previously described^70^. DC maturation was carried out using lipopolysaccharide and IFNγ as reported previously^71^. To label mature DC for in vivo vaccination or in vitro killing assay, mature DCs were washed with 1× PBS twice and then stained with 500 nM of amph-mimotope.

### Immunocompetent B-ALL/lymphoma mouse model

To develop an immunocompetent mouse model for CAR-T cell therapy, we utilized a tumour cell line derived from an *Eμ-Myc* transgenic mouse, which develops B-ALL and Burkitt-like B cell lymphoma-like malignancy^42,72^. *Eµ-Myc* cells were modified to express human CD19 (hCD19) using a retroviral vector. Purification of transduced cells was performed using anti-PE MicroBeads (Miltenyi Biotec) and PE-conjugated anti-human CD19 antibody. To enable monitoring of disease progression, hCD19^+^ *Eµ-Myc* cells were transduced with retroviral vector encoding mCherry and firefly luciferase. Cells were cultured in a medium composed of a 50:50 mix of IMDM with l-glutamine and 25 mM HEPES (Gibco) and DMEM with l-glutamine and sodium pyruvate (Corning), supplemented with 10% FBS and 2-mercaptoethanol to a final concentration of 0.05 mM (Gibco).

All animal work in this model was conducted under the CHOP Department of Veterinary Services (DVR) under an animal protocol approved by the Institutional Animal Care and Use Committee at CHOP in accordance with federal, state and local guidelines. B cell lymphomas were established by i.v. injection of 0.5 × 10^6^ *Eµ-Myc* cells in C57BL/6J or B6(Cg)-*Tyr*^*c*^^−^^*2J*^/J (C57BL/6J albino, Jackson Laboratory) mice after sublethal irradiation (500 cGy X-ray irradiation). On day 4, mice received either mock treatment (untransduced CD45.1^+^ T cells), FMC63-mCAR-T or αFITC-FMC63-mCAR-T cells (2 × 10^6^) intravenously, followed by three rounds of weekly s.c. immunization on both sides of the tail base with amph-mimotope vaccine (10 μg of amph-mimotope mixed with 25 μg CDG in 100 μl PBS, 50 μl each side) or amph-FITC vaccine (10 nM of amph-FITC mixed with 25 μg CDG in 100 μl PBS, 50 μl each side), respectively. When indicated, anti-mouse IL-6 antibody (clone MP5-20F3, BioXcell) was administered intravenously at 8 mg per kg body weight in PBS and dexamethasone (Sigma-Aldrich) was administered intravenously at 0.1 mg per kg body weight in PBS as described before^44^. Disease progression was subsequently monitored every 2–3 days using an IVIS Spectrum fluorescence/bioluminescence imaging system (PerkinElmer) with intraperitoneal administration of 150 mg kg^−1^ d-luciferin K^+^ salt (PerkinElmer, catalogue number 122799). Total photon counts or radiance was quantified using Living Image 4.5. Mice were monitored daily and euthanized at morbidity or as recommended by the veterinarian. The toxicity of therapy was monitored using a toxicity score as described before^44^. A toxicity score of 0 corresponded to active, well-groomed mice, with well-kept hair coat and invisible spine. A score of 1 was assigned to mice with signs of hypomotility, tousled hair coat and a partially visible spine. A score of 2 was given in case of somnolence, rough, dull or soiled hair coat, hunched back with visible spine. Peripheral blood was collected on days 11 and 18. CAR-T expansion and immunophenotyping of CAR-T cells were carried out by flow cytometry. Red blood cells were lysed in ACK lysis buffer (Thermo Fisher) before flow cytometry staining followed by a surface staining for CD45.1 (BV421, clone A20), CD62L (PE-Cy7, clone MEL-14) and CD44 (BV711, clone IM7). The number of cells was determined using CountBright Plus Absolute Counting Beads (Thermo Fisher). The serum level of IFNγ, TNF, IL-10, IL-6 and IL-2 was determined using a bead-based multiplex assay panel, LEGENDplex Mouse Th1 panel (BioLegend), according to the manufacturer’s protocol.

### Immunocompetent B16F10 melanoma mouse model

B16F10 cells were obtained from ATCC and cultured in DMEM with 10% FBS. B16F10 cells were modified to express human CD19 (hCD19) using a lentiviral vector. Purification of transduced cells was performed using anti-PE MicroBeads (Miltenyi Biotec) and PE-conjugated anti-human CD19 antibody. B16F10-hCD19 tumours were established by s.c. injection of 0.5 × 10^6^ hCD19^+^ B16F10 cells into the right flank of C57BL/6 recipient mice in 50 μl saline. Mice received lymphodepletion preconditioning with 500 cGy sublethal irradiation at day 5, and the i.v. infusion of αFITC-FMC63-mCAR-T cells (2 × 10^6^) on day 6, followed with or without two weekly amph-FITC immunizations (10 nmol amph-FITC, 25 μg CDG in 100 μl PBS) as described before^15^. Blood for serum cytokine analysis was collected 6 days after first vaccination.

### Intracellular cytokine staining

Peripheral blood was collected from mice receiving CAR-T or CAR-T plus booster vaccines at day 6 post-vaccination. Peripheral blood (100 µl) was processed in ACK lysis buffer, and PMBCs were resuspended in 100 µl RPMI1640 medium with 10% FBS and 2× Golgi plug (BioLegend). 10^5^ *Eμ-Myc* hCD19^+^ target cells were resuspended in RPMI1640 medium with 10% FBS. Target cells (100 µl) were mixed with 100 µl of PBMCs, transferred to 96-well flat-bottom plates and cultured at 37 °C for 6 h. As a positive control, extra PMBCs from mice receiving CAR-T were combined and cultured with both 1× Golgi plug and cell stimulation cocktail for 6 h. Cells were then resuspended and transferred to a 96-well V-bottom plate for downstream processing. Cells were pelleted and washed once with PBS, and stained with Live/Dead aqua for 15 min in the dark at 25 °C. Cells were pelleted again and surface stained for CD45.1 (PerCP, clone A20) for 20 min on ice followed by 1 wash with flow cytometry buffer. Cells were resuspended in 75 µl of BD Fix/Perm and kept at 4 °C for 15 min, and then washed once by direct filling with 200 µl 1× Perm/Wash (Thermo Fisher). The pellet was resuspended in 50 µl of cytokine antibody cocktail (IFNγ (BV421, clone XMG1.2) at 1:100, TNF (PE-Cy7, clone MP6-XT22) at 1:100) pre-diluted in 1× Perm/Wash buffer, 30 min on ice, then washed once with 1× Perm/Wash buffer and resuspended in 1× flow cytometry buffer for analysis immediately or kept at 4 °C for analysis on a BD Fortessa X-20 flow cytometer the next day.

For intracellular staining from CD19 CAR-T cell-treated NSG mice, splenocytes were resuspended in 200 µl RPMI1640 medium with 10% FBS, Golgi plug (BioLegend) and eBioscience Cell Stimulation Cocktail (Thermo Fisher), transferred to 96-well flat-bottom plates and cultured at 37 °C for 6 h. Cells were then transferred to a 96-well V-bottom plate for downstream processing. Cells were pelleted and washed once with PBS, and then stained with Live/Dead aqua for 15 min in the dark at 25 °C. Cells were pelleted again and surface stained for CD3 (PerCP-eFluor710, clone OKT3) and Myc-tag (AlexaFluor 647, clone 9B11) for 20 min on ice followed by 1 wash with flow cytometry buffer. Cells were resuspended in 75 µl of BD Fix/Perm and kept at 4 °C for 15 min, and then washed once by direct filling with 200 µl 1× Perm/Wash (Thermo Fisher). The pellet was resuspended in 50 µl of cytokine antibody cocktail (IFNγ (BV421, clone 4S.B3) at 1:50, TNF-α (BV605, clone Mab11) at 1:50) pre-diluted in 1× Perm/Wash buffer, 30 min on ice, then washed once with 1× Perm/Wash buffer and resuspended in 1× flow cytometry buffer for analysis immediately or kept at 4 °C for analysis on a BD Fortessa X-20 flow cytometer the next day.

### LN analysis

For LN tissue section imaging, C57BL/6 mice were lymphodepleted and injected with 0.5 × 10^6^ hCD19^+^ *Eμ-Myc* cells. On day 7, mice were adoptively transferred with 2 × 10^6^ FMC63-mCAR-T cells or control T cells, and then vaccinated 1 day later with 10 μg amph-F12-A1 (Vax). Inguinal (draining) LNs were collected on day 15. LNs were flash frozen in tissue cutting medium and sectioned into 10-μm-thick sections on a Leica Cryostat and stored at −80. The sections were fixed in 10% formalin and permeabilized and blocked with Perm/Block solution containing 1% bovine serum albumin and 0.01% Triton-X, and then washed in 1× PBS. The sections were then treated with Fc blocker (Innovex) and stained with antibody solution (1:100 anti-CD3e AF488 (BioLegend 100321), 1:100 anti-B220 AF594 (BioLegend 103254) and 1:75 anti-CD11c AF647 (BD Biosciences 565587)) diluted in Perm/Block buffer in a humidity chamber for 1.5 h. The sections were washed 3 times in PBS and then stained with 300 nM DAPI solution for 5 min. The slides were washed once more and mounted with ProLong Diamond anti-fade solution. The tissue sections were imaged on a Leica Sp8 laser scanning microscope with a 25× objective. Laser power was kept consistent across groups. Shown are representative images of LN sections, with magenta representing B cells, green representing T cells and white representing CD11c DCs. Scale bars represent 200 μm.

### Human B-ALL mouse model and therapeutic studies

All animal work was conducted under an MIT Division of Comparative Medicine Institute Animal Care or CHOP Department of Veterinary Services (DVR), used a committee-approved animal protocol by the Committee of Animal Care at MIT or Institutional Animal Care and Use Committee at CHOP and used a committee-approved protocol in accordance with federal, state and local guidelines. Eight- to twelve-week-old NOD.Cg-*Prkdc*^*scid*^*Il2rg*^*tm1Wjl*^/SzJ (NSG) mice (Jackson Laboratory) were injected with 0.5 × 10^6^ NALM6-Luc cells and randomly assigned into each treatment group. On day 4, leukaemia-bearing NSG mice received either mock treatment or a suboptimal dose of CD19 CAR-T cells (2 × 10^5^ for Fig. 7 or 5 × 10^5^ for Extended Data Fig. 10) intravenously. Twenty-four hours later, one group of CAR-T-treated mice also received amph-mimotope decorated MoDCs (DC-mVax) every 4 days. Leukaemia progression was subsequently monitored every 4–7 days using a Xenogen IVIS fluorescence/bioluminescence imaging system (PerkinElmer) with intraperitoneal administration of 150 mg kg^−1^ d-luciferin K^+^ salt (PerkinElmer 122799). Total photon counts or radiance was quantified using Living Image 4.5. Mice were monitored daily and euthanized at morbidity, >20% weight loss, severe graft versus host disease or as recommended by the veterinarian. To monitor CAR-T cell expansion and persistence, peripheral blood was collected retro-orbitally, and 50 μl from each mouse was used for each flow cytometry analysis. Red blood cells were lysed in ACK lysis buffer (Thermo Fisher) before flow cytometry staining and the total number of PBMCs per microlitre blood in each sample was estimated by cell counting under a microscope or using CountBright Plus Absolute Counting Beads (Thermo Fisher). Spleens were collected from mice receiving CAR-T or CAR-T plus DC-mVax on day 19. Splenocytes were stained with Live/Dead aqua, followed by a surface staining for CD3 (PerCP-eFluor647, clone OKT3), CD4 (PE-Cy7, clone RPA-T4), Myc tag (AlexaFluor 647, clone 9B11), CD45RA (AlexaFluor 488, clone HI100) and CCR7 (PE, clone G043H7) or stained intracellularly for cytokines as described above.

### Data mining and bioinformatic analysis

Single-cell RNA-sequencing data from the bone marrow of human patients with ALL were obtained from GSE134759. Cells with under 500 unique genes detected were filtered, and the data were normalized by library size using the ‘NormalizeData’ function in Seurat (V5) in R 4.4.0. Variable features were selected using the ‘FindVariableFeatures’ function, followed by data scaling with the ‘ScaleData’ function and principal components analysis using ‘RunPCA’. The data were processed for batch correction using Harmony. Clusters were defined using the ‘FindClusters’ function, and two-dimensional embeddings were generated using uniform manifold approximation and projection (UMAP). Differential gene expression analysis was performed using the ‘FindMarkers’ function. Likely malignant B cells were identified as two clusters of B cells that were primarily detected in samples with a diagnosis of ‘Diagnosis’ or ‘Relapse’. A score for co-stimulatory marker expression was computed using the ‘AddModuleScore’ function in Seurat with the following list of genes: *CD80*, *CD86*, *TNFSF9*, *ICOSLG*, *TNFSF4*, *TNFSF18* and *TNFSF14*. Supporting code is available at https://github.com/duncanmorgan/CART-NATBME/blob/main/Figures.ipynb.

### Statistics, selection of animals and justification of sample size

Statistical analyses were performed using GraphPad Prism 8. Animal survival was analysed using log-rank (Mantel–Cox) test. All pair-wise comparisons were analysed by Student’s *t*-test. Multi-group comparisons were carried out using a one-way analysis of variance (ANOVA) with Tukey’s multiple comparisons test. Experiments that involved repeated measures over a time course, such as the total flux, were analysed using an RM (repeated measures) two-way ANOVA based on a general linear model. The RM design included factors for time, treatment and their interaction. Tukey’s multiple comparisons test was carried out for the main treatment effect. *P* values are adjusted to account for multiple comparisons in both one-way ANOVA and RM two-way ANOVA. We determined the size of samples for experiments involving either quantitative or qualitative data as previously reported^73^. On the basis of our previous experience with the animal models and as reported by others^72,74,75^, we consider the therapy as significant if it increases the survival of animals up to 100% within 4 weeks, and ≥5 animals per group is necessary to achieve this goal with 95% confidence interval and at 80% power.

### Reporting summary

Further information on research design is available in the Nature Portfolio Reporting Summary linked to this article.

## Supplementary information

Reporting Summary

## Source data

Source Data Fig. 1 Statistical source data.

Source Data Fig. 2 Statistical source data.

Source Data Fig. 3 Statistical source data.

Source Data Fig. 5 Statistical source data.

Source Data Fig. 6 Statistical source data.

Source Data Fig. 7 Statistical source data.

Source Data Extended Data Fig. 2 Statistical source data.

Source Data Extended Data Fig. 3 Statistical source data.

Source Data Extended Data Fig. 4 Statistical source data.

Source Data Extended Data Fig. 6 Statistical source data.

Source Data Extended Data Fig. 7 Statistical source data.

Source Data Extended Data Fig. 8 Statistical source data.

Source Data Extended Data Fig. 9 Statistical source data.

Source Data Extended Data Fig. 10 Statistical source data.

## Data Availability

Refer to the methods above for specific datasets. Supporting code: https://github.com/duncanmorgan/CART-NATBME/blob/main/Figures.ipynb. Source data are provided with this paper.
