# Supplementary material for: Directed evolution-based discovery of ligands for in vivo restimulation of chimeric antigen receptor T cells
Source: Nat Biomed Eng. 2025 Aug 25;10(4):711–31. doi: 10.1038/s41551-025-01470-0 (PMC13099375; doi:10.1038/s41551-025-01470-0)
Supplement: Supplementary file 1 — Reporting Summary [file 41551_2025_1470_MOESM1_ESM.pdf]

nature portfolio

Corresponding author(s): Darrell Irvine, Leyuan Ma

Last updated by author(s): April 15, 2025

Reporting Summary

Nature Portfolio wishes to improve the reproducibility of the work that we publish. This form provides structure for consistency and transparency in reporting. For further information on Nature Portfolio policies, see our Editorial Policies and the Editorial Policy Checklist.

Statistics

For all statistical analyses, confirm that the following items are present in the figure legend, table legend, main text, or Methods section.

n/a	Confirmed	
☐	☒	The exact sample size (*n*) for each experimental group/condition, given as a discrete number and unit of measurement
☐	☒	A statement on whether measurements were taken from distinct samples or whether the same sample was measured repeatedly
☐	☒	The statistical test(s) used AND whether they are one- or two-sided<br>*Only common tests should be described solely by name; describe more complex techniques in the Methods section.*
☒	☐	A description of all covariates tested
☒	☐	A description of any assumptions or corrections, such as tests of normality and adjustment for multiple comparisons
☐	☒	A full description of the statistical parameters including central tendency (e.g. means) or other basic estimates (e.g. regression coefficient) AND variation (e.g. standard deviation) or associated estimates of uncertainty (e.g. confidence intervals)
☐	☒	For null hypothesis testing, the test statistic (e.g. *F*, *t*, *r*) with confidence intervals, effect sizes, degrees of freedom and *P* value noted<br>*Give P values as exact values whenever suitable.*
☒	☐	For Bayesian analysis, information on the choice of priors and Markov chain Monte Carlo settings
☐	☒	For hierarchical and complex designs, identification of the appropriate level for tests and full reporting of outcomes
☒	☐	Estimates of effect sizes (e.g. Cohen's *d*, Pearson's *r*), indicating how they were calculated

Our web collection on statistics for biologists contains articles on many of the points above.

Software and code

Policy information about availability of computer code

Data collection	GSE134759
Data analysis	Seurat V5, R 4.4.0

For manuscripts utilizing custom algorithms or software that are central to the research but not yet described in published literature, software must be made available to editors and reviewers. We strongly encourage code deposition in a community repository (e.g. GitHub). See the Nature Portfolio guidelines for submitting code & software for further information.

Data

Policy information about availability of data

All manuscripts must include a data availability statement. This statement should provide the following information, where applicable:

- Accession codes, unique identifiers, or web links for publicly available datasets
- A description of any restrictions on data availability
- For clinical datasets or third party data, please ensure that the statement adheres to our policy

GSE134759 accessible on GEO

Research involving human participants, their data, or biological material

Policy information about studies with human participants or human data. See also policy information about sex, gender (identity/presentation), and sexual orientation and race, ethnicity and racism.

Reporting on sex and gender	Human participants are not directly involved in this study. Data were obtained from previously published trail (NCT01626495,NCT02906371, NCT02030847, NCT02030834). Data regarding Sex and Gender are not reported because not investigated as covariate of the correlation between disease burden and expansion.
Reporting on race, ethnicity, or other socially relevant groupings	Human participants are not directly involved in this study. Data were obtained from previously published trail (NCT01626495,NCT02906371, NCT02030847, NCT02030834).
Population characteristics	Human participants are not directly involved in this study. Data were obtained from previously published trail (NCT01626495,NCT02906371, NCT02030847, NCT02030834).
Recruitment	Human participants are not directly involved in this study. Data were obtained from previously published trail (NCT01626495,NCT02906371, NCT02030847, NCT02030834).
Ethics oversight	Human participants are not directly involved in this study. Data were obtained from previously published trail (NCT01626495,NCT02906371, NCT02030847, NCT02030834). The study protocols were approved by the institutional review boards of the University of Pennsylvania

Note that full information on the approval of the study protocol must also be provided in the manuscript.

Field-specific reporting

Please select the one below that is the best fit for your research. If you are not sure, read the appropriate sections before making your selection.

☒ Life sciences ☐ Behavioural & social sciences ☐ Ecological, evolutionary & environmental sciences

For a reference copy of the document with all sections, see nature.com/documents/nr-reporting-summary-flat.pdf

Life sciences study design

All studies must disclose on these points even when the disclosure is negative.

Sample size

We determined the size of samples for experiments involving quantitative data using the following equation:

Sample size = 2 SD2 (Z⍰/2+ Z⍰)2/d2
Standard deviation = from previous studies
Z⍰/2= Z0.05/2= Z0.025= 1.96 (From Z table) at type 1 error of 5%
Z⍰ = Z0.2= 0.842 (From Z table) at 80% power
d = Difference between mean values.

For experiments involving qualitative data, such as animal survival, the following equation was used to estimate required sample size:

Sample size = 2 (Z⍰/2+ Z⍰)2× P (1 – P)/(p1– p2) 2
Z⍰/2= Z0.05/2= Z0.025= 1.96 (From Z table) at type 1 error of 5%
Z⍰ = Z0.2= 0.842 (From Z table) at 80% power
p1 – p2= Difference in proportion of events in two groups
P = Pooled prevalence = (prevalence in case group [p1] + prevalence in the control group [p2])/2

Data exclusions	No data were excluded from the analyses.
Replication	at least 2-3 biological replicates for in vitro experiments, >3 mice were included for each in vivo experiment. Data are reproducible.
Randomization	Mice were randomly allocated into each group. No randomization was performed for experiments other than those involving mice.
Blinding	The investigators were not blinded to group allocation during data collection and/or analysis. Given the objective nature of the measurements and the experimental design, blinding was not deemed relevant to the outcomes of these experiments.

Reporting for specific materials, systems and methods

We require information from authors about some types of materials, experimental systems and methods used in many studies. Here, indicate whether each material, system or method listed is relevant to your study. If you are not sure if a list item applies to your research, read the appropriate section before selecting a response.

Materials & experimental systems

n/a	Involved in the study	
	☒	Antibodies
	☒	Eukaryotic cell lines
☒		Palaeontology and archaeology
	☒	Animals and other organisms
	☒	Clinical data
☒		Dual use research of concern
☒		Plants

Methods

n/a	Involved in the study	
☒		ChIP-seq
	☒	Flow cytometry
☒		MRI-based neuroimaging

Antibodies

Antibodies used

All antibodies listed here were diluted to 0.2mg/ml and used at 1:200 for flow staining

Anti-mouse CD3 (17A2) Alex488 Biolegend 100220; RRID:AB_1732057
Anti-mouse CD45.l (A20) BV421 Biolegend 110732
BRIO: AB_2562563
Anti-mouse CD45.2 (104) BUV737 BD Biosciences 612778; RRID:AB_2870107 Anti-mouse CD8a (53-6.7) BUV395 BD Biosciences 563786; RRID:AB_2732919 Anti-mouse B220 (RA3-6B2) Percp-cy5.5 Biolegend 103222;
RRID: AB_313005
Anti-mouse CDllc (N418) BV785 Biolegend 117335; RRID:
AB_11219204
Anti-mouse CDllb (Ml/70) BV421 Biolegend 101251; RRID: AB_2562904 Anti-mouse CD24 (Ml/69) BUV395 BD Biosciences 744471; RRID: AB_2742259 Anti-mouse MHC II (M5/114.15.2) PE-cy7 Biolegend 107630; RRID:
AB_2290801
Anti-mouse F4/80 (
W20065B) PE Biolegend 111604; RRID: AB_3082990
Anti-human CD80 (
W17149D) APC Biolegend 375404; RRID:
AB_2890817
Anti-human CD86 (
W17233E) APC
Biolegend 381004; RRID: AB_2927984
Anti-human 41BBL (
5F4) APC Biolegend 311506; RRID: AB_2561310
Anti-human OX40L (
11C3.l) PE Biolegend 326308; RRID: AB_2207271
Anti-human ICOSL (
2D3) PE Biolegend 309404; RRID: AB_314770
Anti-Myc tag (9Bll) PE Cell signaling 37395;
Anti-human CD8 (
SKl) APC Biolegend 344722; RRID: AB_2075388
Anti-human CD19 (HIB19) FITC Biolegend 302256; RRID: AB_2564143
Anti-human CD19 (FMC63) In-house
Anti-HA tag (16B12) BV421
Biolegend 682405; RRID: AB_2716037

Validation

The validation of the antibodies was carried out by the manufacturers. The anti-ALK IgG was diluted to 0.2mg/ml and used at 1:200 for flow staining and verified on ALK +/- neuroblastoma cells.

Eukaryotic cell lines

Policy information about cell lines and Sex and Gender in Research

Cell line source(s)

K562, Jurkat, NALM6 and 293 phoenix cells were obtained from ATCC. The NALM6-Luc cell line was a gift from Dr. Michael Birnbaum at MIT. The Eμ-Myc cell line was a gift from Dr. Michael Hemann at MIT.

Authentication

The cell lines obtained from ATCC were not authenticated after their purchase.

Mycoplasma contamination

negative

Commonly misidentified lines
(See ICLAC register)

No commonly misidentified lines were used in the study

Animals and other research organisms

Policy information about studies involving animals; ARRIVE guidelines recommended for reporting animal research, and Sex and Gender in Research

Laboratory animals	Wildtype female C57BL/6 mice (CD45.2+), CD45.1+ congenic mice, B6(Cg)-Tyrc-2J/J (C57BL/6J albino) and NSG mice were purchased from the Jackson Laboratories. All animal studies were carried out following an IACUC-approved protocol following local, state, and federal guidelines. 8-12 weeks old female mice were used in the study. Mice were housed in an animal facility with 12-hour light/12-hour dark cycle with temperatures within 68–72°F and 30–70% humidity.
Wild animals	no wild animals were used in the study.
Reporting on sex	female mice were used
Field-collected samples	no field collected samples were used in the study.
Ethics oversight	All animal studies were carried out following an IACUC-approved protocol following local, state, and federal guidelines. The study was approved by IACUC of the Massachusetts Institute of Technology and the Children's Hospital of Philadelphia.

Note that full information on the approval of the study protocol must also be provided in the manuscript.

Clinical data

Policy information about clinical studies

All manuscripts should comply with the ICMJE guidelines for publication of clinical research and a completed CONSORT checklist must be included with all submissions.

Clinical trial registration	Human participants are not directly involved in this study. Data were obtained from previously published trail (NCT01626495,NCT02906371, NCT02030847, NCT02030834).
Study protocol	Human participants are not directly involved in this study. Data were obtained from previously published trail (NCT01626495,NCT02906371, NCT02030847, NCT02030834)<br>For NCT02030847 the protocol is available here https://clinicaltrials.gov/study/NCT02030847<br>For NCT02030834 the protocol is available here https://clinicaltrials.gov/study/NCT02030834<br>For NCT01626495 the protocol is available here https://clinicaltrials.gov/study/NCT01626495<br>For NCT02906371 the protocol is available here https://clinicaltrials.gov/study/NCT02906371
Data collection	Human participants are not directly involved in this study. Data were obtained from previously published trail (NCT01626495,NCT02906371, NCT02030847, NCT02030834). Data regarding CART expansion and disease burden were collected at the University of Pennsylvania during the clinical trial operations.
Outcomes	Human participants are not directly involved in this study. Data were obtained from previously published trail (NCT01626495,NCT02906371, NCT02030847, NCT02030834).<br>In this study, the primary and secondary outcomes of the trial were not reported; only correlative data between CAR T-cell expansion and disease burden were presented, representing the primary outcomes of our retrospective correlative analysis.

Plants

Seed stocks	N/A
Novel plant genotypes	N/A
Authentication	N/A

Flow Cytometry

Plots

Confirm that:

- [ ] The axis labels state the marker and fluorochrome used (e.g. CD4-FITC).
- [ ] The axis scales are clearly visible. Include numbers along axes only for bottom left plot of group (a 'group' is an analysis of identical markers).
- [x] All plots are contour plots with outliers or pseudocolor plots.
- [x] A numerical value for number of cells or percentage (with statistics) is provided.

Methodology

Sample preparation

Flow cytometry of the yeast:
30x of enriched and induced yeast cells from the 2nd round of positive sorting were pelleted and stained with 30 µl 5 µM control IgG or FMC63 IgG at 4°C for 30 minutes. Yeast was washed twice with 1 ml of 1x PBSA to remove residual antibody. When a plate was used for staining, yeast was washed 3-4 times with 200 µl. The pellet was stained in 50 µl of 1:100 dilution PE-Streptavidin and BV421-HA for 20 minutes on ice. Yeast was washed twice with 1 ml of 1x PBSA prior to flow cytometry sorting (BD FACS Aria), with adjustments made as previously described for using a plate when needed. The top 0.5-1% of the major population based on FMC63 IgG binding was sorted. Usually following this modified protocol, a clearly distinct yeast population could be observed during flow cytometry analysis. 6) FMC63 IgG was used at 0.5µM for the subsequent flow cytometry-based sort. We alternated between streptavidin and anti-biotin antibodies when staining yeast populations for flow cytometry to avoid selecting streptavidin binders. 7) For kinetic sorting, 10x of the library V3 was stained with 50nM biotinylated FMC63 scFv for 30min, washed 2x with 1x PBS and then incubated with 500 nM of non-modified FMC63 IgG for 1 hour or overnight
Flow cytometry of lymph nodes:
Inguinal LNs were extracted and dissociated into single cell suspension for flow cytometry staining for macrophages (MHCII +CD11b+CD11c-F4/80+), cDC1(MHCII+ CD11c+CD11blow/-CD24+) and cDC2 (MHCII+CD11c+CD11b+CD24low/-) as previously described11. To detect amph-mimotope decoration of various lymph node cell populations, 100nM of biotinylated FMC63 IgG was included in the antibody cocktail followed by secondary staining with AlexaFluor 647-streptavidin. For amph-ALK123 mimotope E4, target cells were labeled as described before, stained with biotinylated 100nM ALK123IgG followed by secondary staining with PE-streptavidin.
Flow cytometry of murine blood:
Peripheral blood was collected on days 11 and 18. CAR-T expansion and immunophenotyping of CAR-T cells was carried out by flow cytometry. Red blood cells were lysed in ACK Lysis Buffer (Thermo Fisher) before flow cytometry staining followed by a surface staining for CD45.1 (BV421, clone: A20), CD62L (PE-Cy7, clone: MEL-14), CD44 (BV711, clone: IM7). The number of cells was determined using CountBright Plus Absolute Counting Beads (Thermo Fisher). For NSG mice, peripheral blood was collected retro-orbitally, 50µl from each mouse was used for each flow cytometry analysis. Red blood cells were lysed in ACK Lysis Buffer (ThermoFisher) prior to flow cytometry staining and the total number of PBMCs per microliter blood in each sample was estimated by cell counting under a microscope or using CountBright Plus Absolute Counting Beads (Thermo Fisher). Spleens were collected from mice receiving CAR-T or CAR-T plus DC-mVax on day 19. Splenocytes were stained with live/dead aqua, followed by a surface staining for CD3 (PerCP-eFluor647, clone: OKT3), CD4 (PE-Cy7, clone: RPA-T4), Myc tag (AlexaFluor 647, clone: 9B11), CD45RA (AlexaFluor 488, clone: HI100), CCR7 (PE, clone: G043H7) or stained intracellularly for cytokines as described above
Intracelullar staining:
Peripheral blood (PB) was collected from mice receiving CAR-T or CAR-T plus booster vaccines at day 6 post-vaccination. 100 µl PB was processed in ACK lysis buffer, PMBCs resuspended in 100 µl RPMI1640 medium with 10% FBS and 2X Golgi plug (Biolegend). 105 Eµ-Myc hCD19+ target cells were resuspended in RPMI1640 medium with 10% FBS. 100 µl of target cells was mixed with 100µl of PBMCs, transferred to 96-well flat-bottom plates and cultured at 37°C for 6 hr. As a positive control, extra PMBCs from mice receiving CAR-T were combined and cultured with both 1X Golgi plug and cell stimulation cocktail for 6 hr. Cells were then resuspended and transferred to 96-well V-bottom plate for downstream processing. Cells were pelleted and washed once with PBS, and stained with live/dead aqua for 15 min in the dark at 25°C. Cells were pelleted again, surface stained for CD45.1 (PerCP, clone:A20) for 20 min on ice followed by 1 wash with flow cytometry buffer. Cells were resuspended in 75 µl of BD Fix/Perm and kept at 4°C for 15 min, then washed once by direct filling with 200 µl 1x Perm/Wash (Thermo Fisher). The pellet was resuspended in 50 µl of cytokine antibody cocktail (IFN-γ (BV421, clone:XMG1.2) at 1:100, TNF-α (PE-Cy7, clone:MP6-XT22) at 1:100) pre-diluted in 1x Perm/Wash buffer, 30 min on ice, then washed once with 1x Perm/Wash buffer and resuspended in 1x flow cytometry buffer for analysis immediately or kept at 4°C for analysis on a BD Fortessa X-20 flow cytometer the next day.
For intracellular staining from CD19 CAR-T cell-treated NSG mice, splenocytes were resuspended in 200 µl RPMI1640 medium with 10% FBS, Golgi plug (Biolegend), and eBioscience™ Cell Stimulation Cocktail (ThermoFisher), transferred 96-well flat-bottom plates and cultured at 37°C for 6 hr. Cells were then transferred to 96-well V-bottom plate for downstream processing. Cells were pelleted and washed once with PBS then stained with live/dead aqua for 15 min in the dark at 25°C. Cells were pelleted again, surface stained for CD3 (PerCP-eFluor710, clone: OKT3) and Myc-tag (AlexaFluor 647, clone: 9B11) for 20 min on ice followed by 1 wash with flow cytometry buffer. Cells were resuspended in 75 µl of BD Fix/Perm and kept at 4°C for 15 min, then washed once by direct filling with 200 µl 1x Perm/Wash (Thermo Fisher). The pellet was resuspended in 50 µl of cytokine antibody cocktail (IFN-γ (BV421, clone: 4S.B3) at 1:50, TNF-α (BV605, clone: Mab11) at 1:50) pre-diluted in 1x Perm/Wash buffer, 30 min on ice, then washed once with 1x Perm/Wash buffer and resuspended in 1x flow cytometry buffer for analysis immediately or kept at 4°C for analysis on a BD Fortessa X-20 flow cytometer the next day.

Instrument

Aurora, BF-Fortessa, BD-LSRII

Software	FlowJo
Cell population abundance	at least 10000 events whenever possible
Gating strategy	In general, cells were first gated on FSC-A/SSC-A. Single cells were gated using FSC-H and FSC-A. Further gating for surface and intracellular antigens was performed based on the specific cells and markers to be analysed. Flow-cytometry gating strategies are provided in the Supplementary figures.

☒ Tick this box to confirm that a figure exemplifying the gating strategy is provided in the Supplementary Information.
